# In-house validation of an LC–MS method for the multiplexed quantitative determination of total allergenic food in chocolate

**DOI:** 10.1007/s00216-023-04894-2

**Published:** 2023-08-24

**Authors:** Rosa Pilolli, Antonella Lamonaca, Chiara Nitride, Elisabetta De Angelis, Christof van Poucke, Nathalie Gillard, Anne-Catherine Huet, Marc De Loose, Jean Henrottin, E. C. N. Mills, Linda Monaci

**Affiliations:** 1 Institute of Sciences of Food Production, National Research Council of Italy (ISPA-CNR), Via Giovanni Amendola 122/O, 70126 Bari, Italy; 2 University of Bari Department of Soil Plant and Food Science, Via Giovanni Amendola 165/A, 70126 Bari, Italy; 3 Department of Agricultural Sciences, University of Naples Federico II, Via Università 100, 80055 Portici, Italy; 4 School of Biological Sciences, Division of Infection, Immunity and Respiratory Medicine, Manchester Academic Health Science Centre, Manchester Institute of Biotechnology, University of Manchester, Manchester, UK; 5 Flanders Research Institute for Agriculture, Fisheries and Food, Brusselsesteenweg 370, 9090 Melle, Belgium; 6 CER Groupe, Rue du Point du Jour, 8, 6900 Marloie, Belgium

**Keywords:** Food allergen, Mass spectrometry, Quantitative method, In-house validation, Uncertainty, Conversion factors

## Abstract

Mass spectrometry has been widely accepted as a confirmatory tool for the sensitive detection of undeclared presence of allergenic ingredients. Multiple methods have been developed so far, achieving different levels of sensitivity and robustness, still lacking harmonization of the analytical validation and impairing comparability of results. In this investigation, a quantitative method has been validated in-house for the determination of six allergenic ingredients (cow’s milk, hen’s egg, peanut, soybean, hazelnut, and almond) in a chocolate-based matrix. The latter has been produced in a food pilot plant to provide a real and well-characterized matrix for proper assessment of method performance characteristics according to official guidelines. In particular, recent considerations issued by the European Committee for Standardization have been followed to guide a rigorous single-laboratory validation and to feature the main method performance, such as selectivity, linearity, and sensitivity. Synthetic surrogates of the peptide markers have been used both in native and labelled forms in matrix-matched calibration curves as external calibrants and internal standards, respectively. A two-order of magnitude range was investigated, focusing on the low concentration range for proper assessment of the detection and quantification limits (LOD and LOQ) by rigorous calibration approach. Conversion factors for all six allergenic ingredients have been determined for the first time to report the final quantitative information as fraction of total allergenic food protein (TAFP) per mass of food (µg_TAFP_/g_food_), since such a reporting unit is exploitable in allergenic risk assessment plans. The method achieved good sensitivity with LOD values ranging between 0.08 and 0.2 µg_TAFP_/g_food_, for all ingredients besides egg and soybean, whose quantitative markers reported a slightly higher limit (1.1 and 1.2 µg_TAFP_/g_food_, respectively). Different samples of chocolate bar incurred at four defined concentration levels close to the currently available threshold doses have been analyzed to test the quantitative performance of the analytical method, with a proper estimate of the measurement uncertainty from different sources of variability. The sensitivity achieved resulted in compliance with the various threshold doses issued or recommended worldwide.

**Supplementary Information:**

The online version contains supplementary material available at 10.1007/s00216-023-04894-2.

## Introduction

In the last decades, mass spectrometry (MS) has been successfully applied to the detection and quantification of allergens in foods [1]. In particular, this methodology shows great promise to provide a reference method for food allergen analysis thanks to the unequivocal allergen identification and the intrinsic multiplexing capability. Although the use of synthetic peptides as external calibrants represents the gold standard for absolute protein quantification, in the case of allergenic food determination, it posed the challenge to retrieve mathematical factors to convert the peptide content, accurately reported by an LC–MS method, to a proper unit, which can be considered relevant from the risk assessment point of view. Indeed, the action levels used to describe allergen thresholds are expressed as mg of total allergenic food proteins because the latter are the food fraction triggering the immune adverse reactions [2]. Few investigations have been reported so far, dealing with this critical issue but with limited applicability to only three allergenic foods, i.e., milk, egg, and peanut [3–8]. Both theoretical [3–5] and experimental approaches [6–8] have been explored with different results, sometimes barely comparable due to the limited availability of curated proteomic sequences. In addition, the multitude of MS methods published in the last 10 years targeting multiple allergenic foods have shown several drawbacks and limitations due to the absence of consensus in the reporting units to be included for the final quantitative determination of the total allergenic food protein [9–17]. This also applied to several sensitive MS-based methods that were in-house validated providing as a result the allergen occurrence expressed as a mass fraction of whole allergenic ingredient per food [17].

In this frame, one of the main objectives of the ThRAll (Thresholds and Reference method for Allergen detection) project was to develop a prototype quantitative reference method for the multiple detection of food allergens in hard-to-analyze food matrices [18]. Specifically, chocolate and broth powder were chosen as representative complex foods and six allergenic foods were chosen as targets including cow’s milk, hen’s egg, peanut, soybean, hazelnut, and almond [19–21]. The two model-incurred food matrices were specifically produced for the project objectives in a food pilot plant to mimic real production processes and were carefully characterized in terms of homogeneity and stability to provide a standardized material used for method development and validation [22]. Previous studies aiming at developing multiplex methods for allergen analysis used milk chocolate and dark chocolate as model matrices [23–26], but recoveries of allergenic marker peptides were found to be low and not satisfactory. Further efforts in similar applications disclosed the critical point of proper optimization of extraction, purification, and digestion steps in challenging matrices where proteins may be bound to polyphenols and tannins [8, 27–29]. Our preliminary results about the optimization of sample preparation protocol for chocolate analysis have been recently published [30]. The protocol was developed and tested in two independent laboratories using different triple quadrupole LC–MS platforms to confirm its robustness and reliability.

In this investigation, the in-house validation of the aforementioned method has been accomplished according to official recommendations and guidelines currently available [31–34]. In particular, considerations released by CEN concerning the main analytical requirements for the development of MS-based methods for the determination of protein-derived peptides as allergenic foods markers were taken into consideration [34]. Matrix-matched calibration curves (MMCC) were prepared with synthetic surrogates of prototypic markers and isotopically labelled analogous. The matrix effect on the detection sensitivity was assessed by statistical comparison of MMCC linear regressions and standard calibration curves (SCC). The method sensitivity and linearity were assessed, and detection/quantification limits were calculated according to the most rigorous calibration approach [34]. Here, the experimental approach based on discovery proteomics of the allergenic foods was applied to determine the proportion of different parent proteins in all six targeted foods. The calculated conversion factors were used to determine the absolute content of the six allergenic foods in four incurred chocolate samples added at 2, 4, 10, and 40 µg_TAFP_/g_food_ concentration levels (mass fraction of the total allergenic food proteins in the food matrix). The overall standard uncertainties associated with the measurement of the allergenic food content were estimated combining the contributions of the various sources of variability of the analytical method. The determined content of the allergenic food together with its expanded uncertainty was calculated whenever the reporting quantitative marker/transition resulted equal to or greater than the relevant limit of quantification. Finally, precision was evaluated on the same test samples as repeatability and intermediate precision. The results obtained from the in-house validation have been critically discussed in the following sections.

## Materials and methods

### Chemicals and reagents

Trypsin Gold Mass Spectrometry Grade was purchased from Promega (Milan, Italy). Solvent and other reagents were purchased from Sigma–Aldrich (Milan, Italy) and VWR International PBI (Milano, Italy). Cellulose acetate syringe filters, 5 μm (size 25 mm), were purchased from Sartorius Italy S.r.l. (Muggiò, MB, Italy). Disposable desalting cartridges PD-10 were purchased from Cytiva, GE Healthcare Life Sciences (Milan, Italy). Strata-X polymeric reversed phase (33 µm; 30 mg; 1 mL; 8B-S100-TAK) was purchased from Phenomenex srl (Milano, Italy).

### Synthetic peptide stock solutions

Customized AQUA synthetic peptides were purchased from Thermo Fisher Scientific (Life Technologies, Monza (MI), Italy), as both native sequences (hereafter referred to as “light” peptides) and isotopically labelled analogous on either lysine (+ 8 Da) or arginine residues (+ 10 Da) (hereafter referred to as “heavy” peptides). Light peptides were requested as AQUA QuantPro grade: purity > 97%, formulation 1 nmol aliquot of standard solutions at 5 pmol/μL concentration in 5% (v/v) acetonitrile/water, concentration precision 25–30% (assessed by amino acid analysis). Heavy peptides were requested as AQUA Basic grade: purity > 95%, formulation single lyophilized batch.

A stock solution of light peptides mixture (SLM) was prepared by mixing equimolar amounts of each marker to a final concentration of 312.5 fmol/μL in 5% (v/v) acetonitrile/water. A diluted working solution of such stock (SLM_D) was also prepared at 100 fmol/μL. Each batch of heavy peptides purchased as lyophilized powder was suspended in 5% (v/v) acetonitrile/water at a final concentration of 100 pmol/µL. A stock solution of heavy peptides mixture (SHM) was prepared by mixing equimolar amounts of each marker to a final concentration of 6.25 pmol/μL in 5% (v/v) acetonitrile/water. A diluted working solution of such stock (SHM_D) was also prepared at 625 fmol/μL.

All the prepared stocks were aliquoted according to the need and kept at − 20 °C until their use to avoid repeated freeze/thaw cycles. The working solutions SLM (312.5 fmol/μL), SLM_D (100 fmol/μL), and SHM_D (625 fmol/μL) were used for selectivity experiments and to prepare calibration curves.

### Production of chocolate bars and optimized protocol for sample preparation

The method validation was applied to a model food matrix (chocolate bar) produced within the ThRAll project [18]. Such matrix was produced in a food pilot plant to mimic the real production process [22]. Chocolate was incurred at five nominal concentration levels of cow’s milk, hen’s egg, peanut, soybean, hazelnut, and almond, namely 0, 2, 4, 10, and 40 µg of total allergenic food proteins (TAFP) per g of food matrix µg_TAFP_/g_food_. The chocolate samples were produced as 5 g chocolate chips, individually packed in sealed aluminum laminate and stored at 4 °C until their use. The homogeneity of the samples was assessed by ELISA on 2 g aliquots, and the stability of the test materials along the whole time of method development and validation was also assessed. A full description of the model food matrix production and characterization can be found elsewhere [22].

Blank and incurred chocolate samples were subjected to an optimized sample preparation protocol fully detailed in a recent paper [30] and summarized in the online resource (Fig. S1). Briefly, three chocolate bars (about 15 g) were carefully ground by a laboratory blender, under refrigerated conditions to avoid melting, and sieved by 1 mm mesh. A 2 g aliquot of the ground sample was extracted with 20 mL of a Tris HCl buffer (200 mM Tris·HCl, pH 9.2 with 5 M urea) for 30 min under vigorous stirring at room temperature (250 rpm by an orbital shaker), followed by an in-bath sonication for 15 min. The supernatant was collected after centrifugation and filtered on a 5 µm cellulose acetate syringe filter. The filtered extract was purified by size exclusion chromatography (SEC) on disposable PD10 desalting columns, preconditioned with ammonium bicarbonate 50 mM, according to the producer spin-protocol. A 600 µL aliquot of the eluted fraction was subjected to a 16 h tryptic digestion after thermal denaturation, chemical reduction, and alkylation [30]. The resulting digests were centrifuged and 500 µL aliquots were withdrawn and transferred into clean microcentrifuge tubes for synthetic peptide spiking. All chocolate samples (blank and incurred) were spiked with a fixed amount of heavy peptide mixture (20 µL of SHM_D added to 500 µL of digested matrix) to provide an internal standard for all the peptide markers. Moreover, blank chocolate digests were spiked also with an increasing amount of light peptides (working solutions SLM and SLM_D) for calibration purposes (see “Preparation of standard and matrix-matched calibration curves” for details).

The resulting spiked samples were purified by solid-phase extraction (SPE) on Strata-X® disposable cartridges (see Fig. S1), preconcentrated by a factor of 5, and analyzed by HPLC-MRM.

### Preparation of standard and matrix-matched calibration curves

Matrix-matched calibration curves (MMCC) were prepared by spiking independent aliquots of blank chocolate digest (500 µL) with an increasing amount of light peptide mixture (SLM_D or SLM depending on the sample) and a fixed amount of heavy peptide mixture (20µL of SHM_D). Ten calibration points were prepared at the final concentration of light peptides equal to 0, 0,5, 1, 1,5, 2, 3, 5, 10, 25, and 50 fmol/µL, and the final concentration of heavy peptides equal to 25 fmol/µL. The resulting samples were subjected to SPE and preconcentrated according to the protocol reported in Fig. S1.

Standard calibration curves (SCC) were prepared by spiking an increasing amount of light peptide mixture (SLM_D or SLM depending on the sample) to an ammonium bicarbonate solution simulating the digestion solvent. Seven-calibration points in the range of 1–50 fmol/µL were prepared and subjected to purification by SPE and preconcentration according to the same protocol designed for the chocolate samples.

Both MMCC and SCC were subjected to HPLC-MRM analysis, with three technical replicates, unless otherwise specified. Precision at the lowest detected point (0.5 and 1 fmol/µL) was also assessed by five and ten biological replicates, respectively.

### Analysis of incurred chocolate samples and precision assessment

Test samples of incurred chocolate bars cited in “Production of chocolate bars and optimized protocol for sample preparation” (0, 2, 4, 10, 40 µg_TAFP_/g_food_) were analyzed for precision assessment.

As for the repeatability evaluation of the analytical method, three independent samples (biological replicates) were prepared for each concentration level, and three instrumental replicates (technical replicates) were acquired for each prepared sample. As for the intermediate precision evaluation, further independent samples were prepared at 40 µg_TAFP_/g_food_ level on different days (*t* = 0, 1, 2, 7) by three independent samples/day and by different operators (analysts 1 and 2). In addition, the intermediate precision also was tested at the 4 µg_TAFP_/g_food_ incurring level on two different days (*t* = 0, 7), by three independent samples/day.

### Allergenic ingredient quantification and conversion of the reporting unit

Absolute quantification of the peptide content was performed on averaged L/H ratio values, $\overline{{y }_{0}}$ (intra- and inter-days), provided that the analytical responses were proven not to be significantly different. The reported peptide content, calculated as fmol/µL by MMCC regression parameters ($y=bx+a$), was converted into total allergenic food protein in chocolate (µg_TAFP_/g_food_) according to the simplified Eq. (1):

1 $${x}_{0,\mathrm{TAFP}} \left[{\mathrm{\mu g}}_{\mathrm{ TAFP}}/{g}_{\mathrm{food}}\right]={x}_{0,\mathrm{peptide}} \left[fmol/\mu L\right]* \frac{{cMM}_{\mathrm{protein}}}{{cCF}_{\mathrm{protein}}}* m*d$$

where

*x*_0,peptide_: peptide content determined as $(\overline{{y }_{0}}-a)/b$ by interpolation of the MMCC and reported in fmol/µL.
*cMM*_protein_: centered molar mass (MM) reported in g/mol and calculated as an average of the maximum and minimum MMs retrieved over all sequenced isoforms/variants currently known for the parent protein.
*cCF*_protein_: centered conversion factor (CF) experimentally determined by discovery experiments on allergenic ingredients and calculated as an average of the maximum and minimum CFs calculated over independent samples.
*m*: ${10}^{-5}$, mathematical factor accounting for (i) the matrix-to-solvent ratio 1:10 applied for protein extraction and for (ii) the conversion between prefixes of the International System of units.
*d*: 1.33, dilution factor applied during the digestion protocol.

### Uncertainty estimation

The overall standard uncertainty ($${u}_{{x}_{0}}$$) associated to the determined allergen content ($${x}_{0,TAFP})$$ was calculated combining the five main contributors of the various sources of variability of the analytical method [32]:

1. The standard uncertainty related to the method precision (${u}_{PR}$) calculated as standard deviation over repeated observations divided by $\sqrt{3}$, assuming a rectangular distribution; both technical and biological replicates were considered to include the instrumental variability as well as the overall variability of the sample preparation (extraction, purification, and digestion).
2. The standard uncertainty related to the concentration precision of the synthetic peptides stock solutions (${u}_{SS}$) quoted by the manufacturer by amino acid analysis as 25–30%; for a conservative approach, the maximum reported value (30%) was taken into consideration and the relative standard deviation was divided by $\sqrt{3}$, assuming a rectangular distribution.
3. The standard uncertainty related to the linear least squares regression line (${u}_{RL}$), calculated as
  2 $${u}_{RL}=\frac{{S}_{{~}^{y}\!\left/ \!{~}_{x}\right.}}{b}*{\left[\frac{1}{p}+\frac{1}{n}+\frac{{\left(\overline{{y }_{0}}-\overline{y}\right)}^{2}}{{b}^{2}{\sum }_{i}{\left({x}^{2}-\overline{x}\right)}^{2}}\right]}^{\mathrm{0,5}}$$

where *b* = slope of the regression line; ${S}_{{~}^{y}\!\left/ \!{~}_{x}\right.}$ = standard deviation of regression residuals; *n* = number of calibration points of the MMCC; *p* = number of replicates of the determined sample; $\overline{{y }_{0}}$ = average L/H ratio of the unknown sample ${x}_{0}$; $\overline{x}$ = average concentration of the MMCC range; $\overline{y}$ = average L/H ratio of the MMCC.

4. The standard uncertainty of the molar mass of marker proteins (${u}_{MM}$) calculated considering the variability of the protein sequences (known isoforms/variants) retrieved in UniprotDB. Ranges were calculated as the difference between the maximum and the minimum values and half-ranges divided by $\sqrt{3}$ were calculated as the corresponding standard uncertainties assuming a rectangular distribution.
5. The standard uncertainty of the conversion factor (${u}_{CF}$) experimentally estimated over independent samples of the same allergenic ingredient. Ranges were calculated as the difference between the maximum and the minimum values and half-ranges divided by $\sqrt{3}$ were calculated as the corresponding standard uncertainties assuming a rectangular distribution.

The five contributors were combined into the overall standard uncertainty (${u}_{{x}_{0}}$) by applying the law of error propagation [17]:

3 $${u}_{{x}_{0}}= {x}_{0}* \sqrt{{ ({~}^{{u}_{PR}}\!\left/ \!{~}_{{x}_{0}}\right.)}^{2}+{ ({~}^{{u}_{SS}}\!\left/ \!{~}_{{x}_{SS}}\right.)}^{2} {+ ({~}^{{u}_{RL}}\!\left/ \!{~}_{{x}_{0}}\right.)}^{2}+{ ({~}^{{u}_{MM}}\!\left/ \!{~}_{cMM}\right.)}^{2}+{ ({~}^{{u}_{CF}}\!\left/ \!{~}_{cCF}\right.)}^{2}}$$

and the expanded uncertainty (${U}_{{x}_{0}}$) of the determined content of each allergenic ingredient was calculated with a coverage factor $k=2$, for an approximate level of confidence of 95%, based on a normal distribution of experimental data [32]:

4 $${U}_{{x}_{0}}=k*{u}_{{x}_{0}}$$

### Trueness/recovery experiments

Raw extracts of the six allergenic ingredients (IREs) were prepared under the same experimental conditions described in “Production of chocolate bars and optimized protocol for sample preparation,” at a theoretical concentration of 2 mg_TAFP_/mL_extract_ according to the ingredient protein content reported in a previous investigation [22] and assuming 100% extraction yield. Two grams aliquots of ground blank chocolate bars was collected in 50 mL centrifuge tubes. One of the blank aliquots was spiked with proper amounts (40 µL) of IREs to a final concentration of 40 μg_TAFP_/g_food_. Such sample will be referred to as “spiked before (SB),” hereafter. The SB sample was left open for 1 h at room temperature to allow solvent evaporation and then stored overnight at 4 °C, together with the unprocessed blank sample. After such incubation, both blank and SB were processed in parallel according to the optimized protocol (“Production of chocolate bars and optimized protocol for sample preparation”), with only one additional step for the blank chocolate sample that after elution by SEC was spiked with the same IREs previously described to obtain a final concentration of 40 µg_TAFP_/g_food_. The latter spiked sample will be referred to as “spiked after (SA),” hereafter. SA and SB were digested and purified according to the optimized protocol (“Production of chocolate bars and optimized protocol for sample preparation”). The samples SB and SA were quantified by interpolation of the MMCC, and the percent ratio of the determined content was used as an estimate of the method recovery for each allergenic ingredient on the quantitative marker.

### HPLC-MRM analysis on triple quadrupole

HPLC-MRM analysis was carried out on a UHPLC LX-50 system coupled with a QSight® 220 triple quadrupole mass analyzer (PerkinElmer). Chromatographic separation was performed on a Brownlee SPP Peptide ES-C18 column (2.1 × 150 mm; 2.7 µm; 160 Å) at 30 °C with a binary gradient (A: 0.1% formic acid in water; B: 0.1% formic acid in acetonitrile). The elution gradient applied is the following (flow 0.3 mL/min): 0–3 min constant at 10%B, 3–28 min, from 10% B to 35% B; 28–28.5 from 35% B to 90% B; 28.5–43 min, constant at 90% B; 43–43.5 min from 90% B to 10% B; 43.5–60 min constant at 10% B for column equilibration.

MS acquisition was set up in timed-MRM (multiple reaction monitoring) mode with positive ion analysis, unit resolution in both Q1 and Q3, and with 2 min wide acquisition windows. Ionization source was set as follows: drying gas (nitrogen): 120 (arbitrary units); hot-surface induced desolvation (HSID™) temp: 250 °C; nebulizer gas: 300 (arbitrary units); electrospray V1: 4500; ion source temp: 400 °C. Data processing was carried out using the Simplicity™ 3Q software platform v. 1.6. Instrumental tuning on the specific set of peptide markers was preliminary performed by direct infusion of test samples for the allergenic ingredients (tryptic digests of independent protein extracts for each ingredient). Four transitions were selected for each marker and their isolation/activation voltages were duly optimized (see Table S1 of the online resource). The latter parameters were equally applied to the acquisition of the native and isotopically labelled markers.

Peak areas of each light peptide (L) and its heavy analogous (H) were integrated for all markers and their ratio (L/H) was calculated and used as an analytical signal for any further evaluation.

### Discovery experiments for conversion factor calculation

Discovery experiments were performed on a high-resolution MS platform by data-independent acquisition mode (DIA). A hybrid quadrupole-Orbitrap™ mass spectrometer was used (Q-Exactive Plus by Thermo Fisher Scientific—San Josè, USA) and the chromatographic separation was accomplished on an Acclaim PepMap100, C18 column, (3 μm, 100 Å, 1 × 150 mm). Tryptic digests of independent protein extracts for each allergenic ingredient were prepared at a theoretical protein concentration of 2 mg/mL according to the same protocol used for matrix extraction [30]. Given the high protein content of these test samples, the purification/preconcentration step of the generated peptide mixture was skipped. The tryptic digests were analyzed in Full MS/DIA mode, with six replicates for each sample. Full MS acquisitions were performed as follows: microscan 1, resolution 35 k, AGC target 1e6, maximum injection time 55, range scan 350–1350 m*/z*. The isolation scheme for DIA was set up by Skyline v.21.1 (range 350–1350 m*/z*, isolation width 50 m*/z*, margin 2 m*/z*), and the relevant inclusion list was uploaded on the instrumental method setting further parameters, such as microscan 1, resolution 17.5 k, AGC target 2e5, maximum injection time 50 ms, loop count 22, isolation window 50 m*/z*, NCE stepped 27.30. Raw data were processed by MaxQuant (default workflow set up) for sequence identification and protein-relative quantitation [35]. The searching algorithm was applied against customized databases downloaded from Uniprot (https://www.uniprot.org/, accessed on October 26, 2021). The reference proteomes (RP) were retrieved for five out of six taxonomies of interest:

- *Gallus gallus* (RP: UP000000539, protein count: 43,710, last update Dec 2004),
- *Glycine max* (RP: UP000008827, protein count: 74,863, last update Feb 2013),
- *Bos taurus* (RP: UP000009136, protein count: 37,508, last update Mar 2018),
- *Arachis hypogaea* (RP: UP000289738, protein count: 97,595, last update Jan 2019),
- *Prunus dulcis* (RP: UP000327085, protein count 31,934, last update Jan 2020).

As for the *Corylus avellana* taxonomy (ID 13451), no reference proteome is available to date and all the available entries were downloaded (506 accessions). The experimental approach for CF calculation was equally applied to hazelnut, for consistency, notwithstanding the lack of reference proteome; however, the authors want to highlight that the hazelnut set of CFs must be considered provisional awaiting deeper knowledge of whole proteome.

## Results and discussion

### Selectivity

The selectivity of the method is one of the preliminary characteristics that needs to be assessed during method development and validation to ensure that the measured signal is univocally attributed to the analyte of interest and not influenced by other interfering compounds, causing a bias in the measurement. As for mass spectrometry (MS)–based detection, the selectivity of the overall method is strictly related to the specificity of the peptide markers as well as to the composition of the food matrix that might account for interfering peaks within the selected MRM (multiple reaction monitoring) window, in addition to the chromatographic conditions applied for peptides separation.

According to the CEN guidelines, the specificity of the markers used in this investigation was confirmed—both in silico and experimentally [34]. As for the in silico assessment, sequence alignment with the main protein databases available online was carried out and results were published in previous investigations [36, 37]. However, since such in silico evaluations can provide new results as long as public databases are updated, a further check was also carried out more recently by means of the Protein Prospector tool (MS-Homology tool [38]), searching for 100% identity of tryptic peptides within the last released databases, confirming results previously obtained (see Table 1).

**Table 1:** Summary of protein and peptide markers selected for the MS method. Peptide coding used throughout the paper has been reported. Peptide specificity has been assessed by BLAST search against main databases (NCBInr.2013.6.17, UniProtKB. 2020.09.02, and SwissProt.2021.06.18), with the MS-HOMOLOGY tool available on the portal MS Prospector

Allergenic ingredient	Protein (Uniprot ID)	Allergen	Peptide target residue	Native and isotopically labelled peptide sequence (peptide code)	Peptide specificity Taxonomy (ID number)
Milk/caseinate	αS1-Casein (P02662)	Bos d 9	38–49	FFVAPFPEVFGK (mc-FFV)	*Bos taurus* (ID 9913), *Bubalus bubalis* (ID 89462), *Bos motus* (ID 72004)
				FFVAPFPEVFG(K) (+ 8 Da) (mc-FFV-L)	
	αS2-Casein (P02663)	Bos d 10	130–140	NAVPITPTLNR (mc-NAV)	*Bos taurus* (ID 9913), *Bubalus bubalis* (ID 89462), *Bos motus* (ID 72004), *Bos indicus* × *Bos taurus* (ID 30522)
				NAVPITPTLN(R) (+ 10 Da) (mc-NAV-L)	
Milk/whey	β-Lactoglobulin (P02754)	Bos d 5	108–116	VLVLDTDYK (mw-VLV)	*Bos taurus* (ID 9913), *Bubalus bubalis* (ID 89462), *Capra hircus* (ID 9925), *Ovis aries musimon* (ID 9938), *Ovis aries* (ID 9940)
				VLVLDTDY(K) (+ 8 Da) (mw-VLV-L)	
			100–107	IDALNENK (mw-IDA)	
				IDALNEN(K) (+ 8 Da) (mw-IDA-L)	
Egg/white	Ovalbumin (P01012)	Gal d 2	128–143	GGLEPINFQTAADQAR (ew-GGL)	*Gallus gallus* (ID 9031)
				GGLEPINFQTAADQA(R) (+ 10 Da) (ew-GGL-L)	
			324–340	ISQAVHAAHAEINEAGR (ew-ISQ)	*Gallus gallus* (ID 9031), *Coturnix japonica* (ID 93934)
				ISQAVHAAHAEINEAG(R) (+ 10 Da) (ew-ISQ-L)	
Egg/yolk	Vitellogenin-1 (P87498)	Gal d 6	1874–1884	ATAVSLLEWQR (ey-ATA)	*Gallus gallus* (ID 9031)
				ATAVSLLEWQ(R) (+ 10 Da) (ey-ATA-L)	
	Vitellogenin-2 (P02845)	-	919–927	NIGELGVEK (ey-NIG)	*Gallus gallus* (ID 9031), *Larus argentatus* (ID 35669), *Anas platyrhynchos*, *Patagioenas fasciata monilis* (ID 372326), *Aquila chrysaetos chrysaetos* (ID 223781), *Strigops habroptila* (ID 2489341), *Nestor notabilis* (ID 176057), *Columba livia* (ID 8932), *Phaethon lepturus* (ID 97097), *Pterocles gutturalis* (ID 240206), *Charadrius vociferous* (ID 50402)
				NIGELGVE(K) (+ 8 Da) (ey-NIG-L)	
Peanut	Cupin (O82580)	Ara h 3	342–354	SPDIYNPQAGSLK (p-SPD)	*Arachis hypogaea* (ID 3818)
				SPDIYNPQAGSL(K) (+ 8 Da) (p-SPD-L)	
			355–365	TANDLNLLILR (p-TAN)	*Arachis hypogaea* (ID 3818), *Arachis duranensis* (ID 130453)
				TANDLNLLIL(R) (+ 10 Da) (p-TAN-L)	
Soybean	Glycinin (P04776)	Gly m 6	411–423	VLIVPQNFVVAAR (s-VLI)	*Glycine max* (ID 3847), *Glycine* subgen. Soja (3848)
				VLIVPQNFVVAA(R) (+ 10 Da) (s-VLI-L)	
			401–410	VFDGELQEGR (s-VFD)	
				VFDGELQEG(R) (+ 10 Da) (s-VFD-L)	
Hazelnut	11S seed storage Globulin (A0A0A0P7E3)	Cor a 9	339–348	ADIYTEQVGR (h-ADIY)	*Corylus avellana* (ID 13451), *Carpinus fangiana* (ID 176857)
				ADIYTEQVG(R) (+ 10 Da) (h-ADIY-L)	
			462–476	ALPDDVLANAFQISR (h-ALP)	
				ALPDDVLANAFQIS(R) (+ 10 Da) (h-ALP-L)	
Almond	Amandin, 11S Globulin (E3SH28)	Pru du 6	493–505	TEENAFINTLAGR (a-TEE)	*Prunus dulcis* (ID 3755), *Prunus persica* (ID 3760)
				TEENAFINTLAG(R) (+ 10 Da) (a-TEE-L)	
			388–394	ADIFSPR (a-ADIF)	
				ADIFSP(R) (+ 10 Da) (a-ADIF-L)	

In addition, in order to disclose potential interferences from the matrix itself accounted by isobaric coeluting compounds, an analysis of blank chocolate matrix spiked with isotopically labelled peptides was performed to evaluate the degree of interferences in the measured signals and hence to confirm experimentally the selectivity. No significant interferences were detected under the optimized chromatographic separation conditions for the acquired transitions (see Fig. S2). However, special attention was paid to the chromatographic separation of the peptide mw-IDA, to avoid overlapping of the specific signals with an intense interfering band affecting both transitions 458.7/688.4 and 458.7/504.2 (see Fig. S2).

### Calibration and linearity

Absolute quantification of allergenic ingredients in MS-based methods can be achieved by means of an external calibrant, such as a mixture of natural synthetic peptides, a commercially available standard protein, the whole ingredient, or a reference material. Depending on the selected calibrant, the complexity of the pipeline and the overall uncertainty associated with the measurement can change. Here, synthetic surrogates of the selected markers were applied as calibrants and isotopically labelled analogous of the peptide markers were included, as well (see Table 1). These latter were used as an internal standard for each analytical run and applied to convert the measurement of normalized peak area (ratio light-to-heavy peptide form) obtained from MRM acquisition into peptide concentration. Both light and heavy synthetic peptides were added to a fixed amount of blank chocolate digest to obtain matrix-matched calibration curves (MMCC), as recommended by the CEN guidelines to compensate for matrix effects [34]. The spiking of calibrants and internal standards were performed before the purification step by solid-phase-extraction (SPE), in order to normalize the analytical signals and minimize the contribution of the peptide recovery from SPE and of the instrumental fluctuation (chromatographic separation and/or ionization efficiency) to the overall variability.

For MMCC preparation, aliquots of the blank chocolate digest were added with an increasing amount of light peptide mixture and a fixed amount of heavy peptide mixture according to what was described in “Preparation of standard and matrix-matched calibration curves.” In particular, a wide concentration range was taken into consideration for the calibration curve covering two orders of magnitude with a total of ten calibration points, including the blank sample, which exceeded significantly the minimum of five points prescribed by the CEN guidelines [34]. Most of the calibration levels were intentionally selected in the first part of the range, up to 5 fmol/µL, to properly characterize the method performance at the lowest responses.

As the first step, regression lines were calculated on the whole concentration range, with a consistent detection of all markers at the selected levels, except for the egg white peptides ew-ISQ and ew-GGL peak that were detected only starting from the 1 fmol/µL sample (see Table 2). Fitting parameters proved a good linearity of the response (*R*^2^ ≥ 0,993) within the investigated range with a variable sensitivity depending on the specific allergenic ingredient (see Table 2). According to the sensitivity calculated as the slope of the calibration curve, one marker was selected as the quantitative reporter (QTM, quantitative marker) for each allergenic ingredient (two in the case of milk and egg, for partial food formulation), while the second marker was monitored for confirmative purposes (QLM, qualitative marker).

**Table 2:** Summary of the method sensitivity performances for the six allergenic ingredients calculated by matrix-matched calibration curves with synthetic peptides (normalized signal of native peptide by isotopically labelled one)

Allergenic ingredient (QTM^a^)	Retention time (RSD%) [min]	Calibration range [fmol/µl]	Method sensitivity (whole range)		
			Slope	SD slope	*R*^2^
Milk caseinate (mc-FFV)	25.19 ± 0.19 (0.8%)	0.5–50	0.02121	0.00012	0.999
Milk whey (mw-VLV)	13.25 ± 0.12 (0.9%)	0.5–50	0.0259	0.0002	0.998
Egg white (ew-ISQ)	4.4 ± 0.2 (5%)	1–50	0.0534	0.0009	0.996
Egg yolk (ey-ATA)	18.37 ± 0.16 (0.9%)	0.5–50	0.0597	0.0009	0.993
Peanut (p-TAN)	20.62 ± 0.17 (0.8%)	0.5–50	0.0456	0.0003	0.998
Soybean (s-VLI)	19.67 ± 0.17 (0.9%)	0.5–50	0.0340	0.0005	0.994
Hazelnut (h-ALP)	22.03 ± 0.18 (0.18%)	0.5–50	0.0391	0.0004	0.996
Almond (a-TEE)	16.80 ± 0.15 (0.9%)	0.5–50	0.0431	0.0003	0.999

^*a*^*QTM* quantitative marker

General effect of matrix composition on the detection was evaluated by comparing the MMCC with standard curves obtained by analyzing the synthetic peptide mixtures in simple solutions simulating the digestion buffer. Such standard solutions prepared in the same concentration range of the MMCC were purified and preconcentrated by SPE according to the protocol already described for the chocolate samples and subjected to HPLC-MRM analysis. Similarly, the light-to-heavy ratios were calculated, and “standard” calibration curves (SCC) were built in order to provide a set of data very comparable with the MMCC, besides the presence of the chocolate background. The evaluation of the matrix effect was then carried out by a statistical comparison (Student’s *t*-test) of the slopes of the two regression lines MMCC and SCC. Comparing the slopes of two regression lines is a quite common task in analytical laboratories; however, the literature differs in how to calculate the pooled standard error for the *t*-test statistic, and in this case, we referred to the review of Andrade and Estévez-Pérezi from 2014 [39]. In order to select and apply the proper *t*-test hypothesis, a first preliminary screening of the similarity of the regression variances (residual standard deviations, *S*_y/x_) for MMCC and SCC was carried out by the Fisher–Snedecor’s *F*-test, namely dividing the largest by the lowest variance and comparing it to unity (one-tail test). For all the QTMs, the regression variances of MMCC and SCC resulted not significantly different, at a 1% significance level, allowing the pooling of the error variances weighted by their degrees of freedom, for *t*-test execution. The calculated *t*-test statistic proved that the slopes of the regression lines MMCC and SCC were significantly different at a 95% confidence level for all the quantitative markers except for ew-ISQ and mw-VLV. Such results confirmed the relevance of preparing MMCC for the proper absolute quantification of residual allergenic ingredients in complex food matrixes such as chocolate bar.

As already mentioned, special attention was paid to characterize the method performance in the low calibration range, the latter being more relevant to trace back even little amount of unintended contamination. Six calibration points were placed in the range 0.5–5 fmol/µL, and such data set was used to calculate the limits of detections (LOD) and quantification (LOQ). By definition, the detection limit is the smallest concentration of analyte in a test sample that can reliably be distinguished from the blank. Despite the apparent clarity of its definition, the experimental evaluation of the LOD has been debated for a long time; in fact, independent research groups still commonly use different approaches. Our opinion is to calculate LODs and LOQs by the calibration approach considering the variability over the calibration range investigated (standard deviation (SD) of the intercept or of the residuals) or over ten independent samples at the lowest concentration level, to avoid over-optimistic conclusions [1]. In this investigation, we calculated the LOD and LOQ as 3 and 10 times, respectively, the SD of the intercept divided by the slope of the calibration curve. The method reported very good and homogeneous performances for five out of six allergenic ingredients (milk, peanut, soybean, hazelnut, and almond) with LOD values ranging between 0.1 and 0.2 fmol/µL, whereas a slightly higher limit was calculated for ew-ISQ marker up to 0.9 fmol/µL (see Table 3).

**Table 3:** Summary of the method precision at the lowest detected point and calculation of the limits of detection and quantification by calibration approach in the low concentration range (≤ 25 fmol/µL for ew-ISQ and ≤ 5 fmol/µL for all the other markers) of the matrix-matched calibration curves (regression model *y* = *b*x + *a*, *SDa* standard deviation of the intercept)

Allergenic ingredient (QTM^a^)	Precision at the lowest detected point (LDP)	Limit of detection (LOD) = $3*\frac{{SD}_{a}}{b}$ Limit of quantification (LOQ) = $10*\frac{{SD}_{a}}{b}$			
		RU: peptide concentration [fmol/µL]		RU^b^: total protein of allergenic ingredient in matrix [µg_TAFP_/g_food_]	
		LOD	LOQ	LOD	LOQ
Milk caseinate (mc-FFV)	2%	0.11	0.4	0.08	0.3
Milk whey (mw-VLV)	4%	0.13	0.4	0.16	0.5
Egg white (ew-ISQ)	12%	0.9	3	1.1	4
Egg yolk (ey-ATA)	20%	0.2	0.7	14	50
Peanut (p-TAN)	11%	0.10	0.3	0.14	0.5
Soybean (s-VLI)	12%	0.19	0.6	1.2	4
Hazelnut (h-ALP)	17%	0.2	0.7	0.2	0.7
Almond (a-TEE)	7%	0.16	0.5	0.17	0.6

^*a*^*QTM* quantitative marker

^b^The conversion of the reporting units (RU) was performed by applying the factors reported in Table S3

As a further step, we tested the method in terms of precision at the lowest detected points (LDP); indeed, besides the analytical approach followed to calculate the detection/quantification limits, the precision at LDP represents a very crucial point for the method reliability assessment. The performance was very satisfying with a relative SD ≤ 20% for all the QTMs (see Table 3).

In order to provide a complete overview of the method sensitivity, we also calculated the limits LOD and LOQ with alternative estimates of the SD from (i) the residuals SD of the linear regression and from (ii) the signal SD of the lowest detected point. Such data have been appended in Table S2 of the online resource. As a general comment, the LOD/LOQ estimates based on the intercept or on the LDP SDs are often very comparable, confirming that can be equally applied; whereas the limits calculated on the regression residuals resulted to be always overestimated, likely due to the more rigorous analytical approach, which encompasses the variance overall the regression line.

### Absolute quantification

#### Reporting units and conversion factors

The application of synthetic peptides as external calibrants for absolute quantification relies on the assumption that the parent protein is completely digested in the sample with an equimolar release of the marker peptides and that these latter are stable during digestion. In this analytical method, both hypotheses have been tested and confirmed in the method development phase [30]. As such, this approach can provide an accurate quantification of the amount of marker peptide in a sample. However, in order to convey usable information from the risk assessment point of view, it needs conversion of the peptide reporting units (RUs) first to mg of parent protein and then to mg of total allergenic food protein (TAFP) in the analyzed matrix. The design and application of mathematical factors to convert from peptide reported to TAFP require several information, some of these easily achievable, because strictly related to the sample preparation protocol (i.e., matrix-to-solvent ratio and dilution factors throughout the workflow), others much more critical. In particular, two main pieces of information, namely the molar mass (MM) of the parent protein and its relative abundance in the total protein profile of the allergenic ingredient (here referred to as conversion factor CF), may represent the main source of uncertainty of the method.

As for the protein molar mass, it is important to consider that the peptide markers often are not unique to a parent protein but are shared across protein isoforms/variants. This should be accounted for in the uncertainty budget and an averaged MM among isoforms should be preferentially used, for a higher representativeness of the values. These considerations were firstly reported in a recent investigation by Martinez-Esteso et al. [3] concerning the milk determination in cookies, and here we decided to apply a similar approach to all six allergenic ingredients of interest. Noteworthy, milk is probably the most widely characterized food ingredient, whereas only partial or very limited information was retrieved for the other five ingredients (egg, peanut, soybean, hazelnut, almond). Nevertheless, all available accessions of protein markers encoding the specific signature peptides were collected, to the best of the current knowledge (see Table S3 of the online resource). The mean between the maximum and minimum MMs (cMM) and the halfwidth of such range divided by $\sqrt{3}$ were calculated for mathematical conversions of the RUs and uncertainty estimates (${u}_{MW}$), respectively (see “Allergenic ingredient quantification and conversion of the reporting unit” and “Uncertainty estimation” and Table S3 of the online resource). Noteworthy, the lower relative standard uncertainties (≤ 0.6%) obtained for the MM of marker proteins other than milk proteins did not reflect higher reliability of the results, but mainly a limited knowledge about protein isoforms/variants.

The second critical information required for the conversion of the RUs is the CF. This information can be retrieved either by a theoretical approach [3–5] or by an experimental approach [6–8]. The theoretical approach requires qualitative and quantitative knowledge of protein profiles of the allergenic food ingredient and provides averaged estimates, which are more likely to be widely representative of the natural variability. However, such detailed knowledge is currently available only for cow’s milk, hen’s egg, and partially for peanut; therefore, the theoretical approach is not affordable yet for most of the major allergenic ingredients. Differently, the experimental approach relies on discovery MS-based experiments carried out on the specific ingredient used in method development to determine the proportions of different parent proteins by spectral counting. Some concerns might rise when the obtained values are used to measure multiple allergenic ingredient sources that may differ in the ratios of their constituent proteins, still, testing a set of allergenic foods from different suppliers might be a solution to obtain more representative experimental CFs. The feasibility of the experimental approach also relies on the availability of well-annotated and curated proteomic sequences, and, as such, can change and improve over time, if new proteomes are collected, curated, and shared on the public domain.

Starting from this background and taking into consideration the current availability of reference proteomes for five out of six allergenic ingredients (see “Discovery experiments for conversion factors calculation”), in this investigation, an experimental approach for conversion factor calculation was performed for consistency.

Briefly, small aliquots of each allergenic ingredient used for the production of the incurred chocolate bar have been extracted with the same buffer of the method under development and digested by trypsin enzyme. The peptide pools were analyzed by high-resolution MS and raw data were processed via software for protein-relative quantitation (see “Discovery experiments for conversion factors calculation” for details). The data set acquired for each allergenic ingredient was searched against its own reference proteome, allowing protein quantification based only on unique and razor peptides. The resulting list of proteins and peptides was browsed for the selected markers. The intensities assigned to the target protein, as well as to any isoform/variant available encrypting the same peptide marker, were divided by the sum of intensities of all the identified proteins. Such a ratio was assumed as an experimental estimate of the protein-relative abundance in the allergenic ingredient. Whenever the peptide marker was detected in more than one isoform/variant, each identified by at least one unique peptide, the sum of the relative abundance of all accessions was considered for CF calculation. The same mathematical calculations were applied to all replicates and averaged CFs as well as their uncertainties (${u}_{CF}$, *k* = 1) were reported in Table S3 of the online resource. It deserves to be noted that very limited knowledge is currently available for hazelnut (see “Discovery experiments for conversion factors calculation”) and this could seriously affect the reliability of the resulting CFs experimentally calculated. However, in the absence of any feasible alternative to calculating conversion factors for hazelnut (neither theoretical nor experimental), we decided to process the discovery data obtained from hazelnut likewise the other allergenic ingredients, although aware of the uncertainty of this specific data set, as a provisional solution, likely to be updated in a near future.

To the best of our knowledge, this represents the first investigation in which CFs are calculated and applied to the absolute quantification of soybean, almond, and hazelnut; therefore, a critical comparison with previous investigations carried out by an independent research group cannot be accomplished. However, some comments and conclusions can be drawn for milk, egg, and peanut by comparing our results with previous achievements from the literature. As for milk, our experimental approach reported a CF of 0.374 with an ${u}_{CF}$ of 0.009 (*k* = 1) for the αS1-casein, here selected as quantitative reporting parent protein. Interestingly, this value did not differ significantly from the theoretical value calculated by Martinez-Esteso et al. [3], for the same marker protein, namely 0.366 with an ${u}_{CF}$ of 0.046 (*k* = 1). On the contrary, our experimental approach resulted in an overestimation of the β-lactoglobulin CF, equals to 0.193 with an ${u}_{CF}$ of 0.002 (*k* = 1), compared to the theoretical value of 0.102 with an ${u}_{CF}$ of 0.010 (*k* = 1) [3]. Noteworthy, the same overestimation of β-lactoglobulin CF by the experimental approach was observed in the paper from Parker et al. [6] in 2015, reporting a CF of 0.178 (no uncertainty was assigned to CF value; therefore, no proper statistical comparison can be afforded). This suggested that for the experimental approach, the result reliability strictly depends on specific protein properties (extractability, digestibility, and ionization efficiency of tryptic peptides). As for egg proteins, no theoretical calculation of CF and relevant standard uncertainty have been proposed yet in the literature; however, good knowledge about the natural variability of egg composition and protein profile is available. Therefore, we can afford to apply the same approach proposed by Martinez-Esteso et al. [3] to egg proteins resulting in a theoretical CF for ovalbumin of 0.31 with an ${u}_{CF}$ of 0.03 (*k* = 1) (all the details for this calculation were reported in the online resource Table S4). Also, in this case, our experimental approach provided an overestimation for ovalbumin CF 0.468 with an ${u}_{CF}$ of 0.003 (*k* = 1), which resulted in an agreement with the experimental CF of 0.421 reported by Parker et al. [6] in 2015. Similarly, the CFs for Ara h 3 calculated from both the experimental approaches resulted very close (0.695 with an ${u}_{CF}$ of 0.006 (*k* = 1) against 0.624 [6]).

The relative standard uncertainties of CF (${u}_{CF,rel}$) calculated for all allergenic ingredients ranged between 1 and 8%, with the highest values assigned to milk (5%) and soybean (8%) CFs (see Table S3 in the online resource). It deserved to be noted that according to its definition, such ${u}_{CF,rel}$ accounts only for the variability related to the analytical method applied for the CF experimental estimation and does not contain any information about the natural variability of the protein profile of the allergenic ingredient. This critical point justifies the lower uncertainty here calculated for αS1-casein (5%) against the reported value of 13%, accounting for the natural variability of cow milk as defined in the paper from Martinez-Esteso et al. [3].

Considering the matrix-to-buffer ratio of 1:10, the dilution factor applied throughout the sample preparation protocol, and the proper prefixes of the international system units, the final conversion from peptide concentration to TAFP in chocolate (µg_TAFP_/g_food_) was accomplished (see “Allergenic ingredient quantification and conversion of the reporting unit” for equations). For the sake of clarity, the conversion factors applied to each reported peptide marker are itemized in Table S3, and these were applied to convert the LOD/LOQ values previously commented, in the µ_TAFP_/g_food_ reporting unit (see Table 3).

#### Absolute quantification of incurred test samples

The quantitative performance of the method under validation was assessed on four test samples prepared within the consortium in a food pilot plant and well characterized in terms of homogeneity and stability [22]. Such samples were incurred chocolate bars (ICB) at defined levels of each allergenic ingredient, namely 2, 4, 10, and 40 µg_TAFP_/g_food_, calculated as a mass fraction of the total protein of the allergenic ingredient in the food matrix. Both biological and technical replicates were analyzed for each concentration level and the recorded analytical signal was used to report for the peptide marker content by interpolation of the MMCC. The determined peptide content was converted into the total protein of the allergenic ingredient in the food matrix according to Eq. (1). The detection of the allergenic ingredients in ICB was considered successful whenever at least two transitions for at least two peptides were detected and quantified above the specific LODs. The quantification of the ingredient content was accomplished whenever the reporting quantitative marker/transition resulted in greater or equal to the relevant LOQ.

The overall standard uncertainty to each determined content was calculated according to Eq. (3) combining five main contributors as sources of variability related to (i) the method precision (${u}_{PR}$), (ii) the concentration precision of the synthetic peptide stock solutions (${u}_{SS}$), (iii) the linear least squares regression line (${u}_{RL}$), (iv) the molecular weight of protein markers (${u}_{MM}$), and (v) the conversion factors (${u}_{CF}$). For the sake of clarity, in Fig. S2, the five contributors were reported as relative standard uncertainty (${u}_{i,rel}$) for each test sample (ICB 2–40 µg_TAFP_/g_food_). Noteworthy, the uncertainties ${u}_{SS, rel}$, ${u}_{MM,rel}$, and ${u}_{CF,rel}$ equally counted on all the tested samples, whereas both the ${u}_{RL, rel}$ and the ${u}_{PR, rel}$ resulted sample-dependent. In particular, the ${u}_{RL, rel}$ disclosed an inverse proportionality with the incurring level for all tested markers. Moreover, the high number of replicates (*p* value in Eq. 2) prepared for the 40 µg_TAFP_/g_food_ ICB sample, which is generally close to the upper limit of the calibration range, also contributes to keeping low the ${u}_{RL, rel}$ at this level. In addition, the ${u}_{PR, rel}$ resulted in sample-dependent but no general trend was observed, because such uncertainty value was affected by several factors, such as the specific marker sensitivity and the number of biological and technical replicates averaged for peptide content determination, which differed over the tested samples. All these results and considerations suggested that drawing general conclusions about the main contributors of the overall method uncertainty is a quite challenging task because it depends on a myriad of factors including the operator, the calibration range, the number of replicates, the sensitivity of the specific marker, and the knowledge about the protein profile of the allergenic ingredient. Therefore, taking into consideration all contributors whenever possible would be the best conservative approach, for an utmost confident dissertation.

**Fig. 1:**
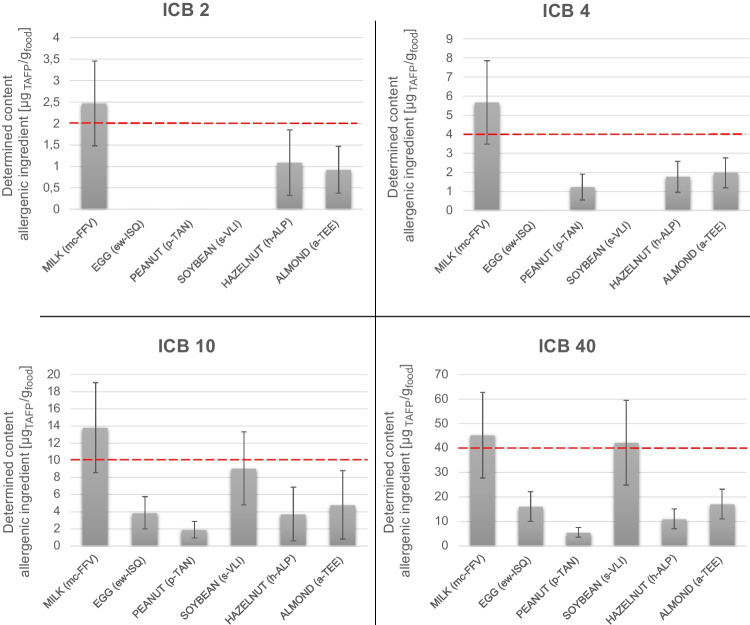
Quantification results with relevant combined and expanded uncertainty (*k* = 2) obtained for incurred chocolate samples (ICB) at four concentration levels (2, 4, 10, 40 µg_TAFP_/g_food_). The determined concentrations were reported as mass fraction of the total allergenic food protein per food matrix by conversion of the reporting units according to the factors reported in Table S3

Finally, the expanded uncertainties were calculated for all quantitative markers/transitions by considering a coverage factor $k=2$, for an approximate confidence level of 95% [32]. The determined content of each allergenic ingredient together with its expanded uncertainty was reported in Fig. 1 and Table S5. In summary, the method succeeded in the quantification of milk, hazelnut, and almond at all the tested concentration levels of ICB samples (2–40 µg_TAFP_/g_food_). Satisfactory results were proved also for peanut, where quantification was accomplished starting from the 4 ppm ICB. Slightly lower sensitivity was displayed for egg and soybean where only the two highest ICB (10–40 µg_TAFP_/g_food_) were quantified.

#### Precision

Repeatability was assessed within the same laboratory ICB at all concentration levels (2–40 µg_TAFP_/g_food_). Three independent samples were prepared by the same operator within the same day and under the same conditions and analyzed by LC-MRM with three technical replicates. The light-to-heavy ratios of each quantifier marker were compared by one-way ANOVA at a 95% confidence level and the three biological replicates resulted not to be significantly different at each concentration level, thus allowing the pooling of all data within the same day. The intra-day coefficient of variation (CV%) resulted very satisfactory being always lower than 15% for all quantitative markers, not showing any particular correlation with the concentration levels tested (see Table 4). Concerning the relative contribution of the instrumental analysis (IA) and the sample preparation (SP) to the overall variance, as a general trend, it was observed that IA was the main contributor at the lowest detected levels, namely 2 µg_TAFP_/g_food_ ICB for milk (caseinate), hazelnut and almond, 4 µg_TAFP_/g_food_ ICB for peanut, and 10 µg_TAFP_/g_food_ ICB for egg (white) and soybean. On the contrary, the SP contribution became prevalent at the highest tested concentration (ICB 40 µg_TAFP_/g_food_) for all QTM except for egg and soybean (see Table 4), in agreement with the lower sensitivity achieved for these two allergenic ingredients.

**Table 4:** Evaluation of the method repeatability and intermediate precision: relative standard deviation (CV%) of the determined peptide content (fmol/µL) in incurred chocolate bar samples and relative contribution of “sample preparation (SP),” “instrumental analysis (IA),” “day of analysis (DA),” and “analyst (A)” to the total variance

QTM	Sample		Repeatability			Intermediate precision			
			Total CV%	Relative weight		Total CV%	Relative weight		
				IA	SP		SP	DA	A
Milk caseinate (mc-FFV)		ICB 2	3%	77%	23%	-	-	-	-
		ICB 4	2%	84%	16%	11%	35%	65%	nt^a^
		ICB 10	15%	3%	97%	-	-	-	-
		ICB 40	8%	8%	92%	8%	71%	27%	1%
Milk whey (mw-VLV)		ICB 40	12%	12%	88%	29%	38%	28%	34%
Egg white (ew-ISQ)		ICB 10	13%	79%	21%	-	-	-	-
		ICB 40	12%	65%	35%	15%	38%	23%	39%
Egg yolk (ey-ATA)		ICB 10	4%	90%	10%	-	-	-	-
		ICB 40	10%	72%	28%	13%	38%	29%	34%
Peanut (p-TAN)		ICB 4	7%	56%	44%	6%	59%	41%	nt^a^
		ICB 10	5%	97%	3%	-	-	-	-
		ICB 40	6%	21%	79%	9%	54%	46%	0%
Soybean (s-VLI)		ICB 10	6%	100%	0%	-	-	-	-
		ICB 40	13%	69%	31%	11%	55%	41%	3%
Hazelnut (h-ALP)		ICB 2	4%	77%	23%	-	-	-	-
		ICB 4	8%	56%	44%	5%	73%	27%	nt^a^
		ICB 10	8%	69%	31%	-	-	-	-
		ICB 40	6%	43%	57%	14%	42%	42%	16%
Almond (a-TEE)		ICB 2	8%	94%	6%	-	-	-	-
		ICB 4	10%	41%	59%	6%	100%	0%	nt^a^
		ICB 10	5%	93%	7%	-	-	-	-
		ICB 40	8%	17%	83%	8%	72%	17%	11%

^*a*^*nt* no test performed for this variable

In addition, the intermediate precision was evaluated at two out of four concentration levels available for ICB samples, selected as low-medium (4 µg_TAFP_/g_food_) and high (40 µg_TAFP_/g_food_) test samples, still covering one order of magnitude in concentration. Both the operator and day of analysis were changed for this set of experiment. Again, the mean values obtained under different conditions were compared by a one-way ANOVA test at a 95% confidence level, turning out not to be significantly different. The acquired data were then averaged providing very promising results with CVs always lower than 15% except for the β-lactoglobulin reporting marker (29%). In particular, the intermediate precision resulted in the 5 to 11% range at the 4 ppm tested level and in the 8 to 15% range at the 40 ppm tested level (see Table 4). As for the relative contribution of different factors on the overall variance, the prevalent sources resulted in the SP and the day of analysis, whereas the operator seemed to be always a lesser contributor except for the egg and soybean ingredients, where the weights seemed to be equally distributed among the three factors.

#### Sample stability over time

Collateral to the precision assessment described in the previous paragraph, an ad hoc experiment was designed to evaluate the stability of the analytical samples produced over a short time period (3 days) and provide a full description of the method performance characteristics.

In particular, such evaluation was carried out on a set of independent ICB samples (40 µg_TAFP_/g_food_) prepared in triplicate on the same day and for three different days (total of nine biological samples). Each sample was analyzed three times on the same day (technical replicates on day 0) and then analyzed again on the two following days, keeping the samples refrigerated at + 8 °C (two technical replicates recorded on days 1 and 2, respectively). The averaged values calculated within the same day and along the 3 days for each quantitative marker were compared by *t*-test at a 5% significance level. As a result, the means calculated for all the markers in each independent sample were proved not to be significantly different in all the independent samples prepared, thus confirming the stability of the samples over 48 h under controlled refrigerated conditions.

### Trueness and recovery experiments

Trueness of the analytical method was assessed by recovery calculation on spiked samples according to CEN guidelines [34]. In particular, blank samples were spiked with protein extract of the allergenic at different stages of the sample preparation protocol for comparative purposes (“spiked before” SB and “spiked after” SA samples, see “Trueness/recovery experiments” for details). The SB and SA samples were quantified by interpolation of the MMCC and the percent ratio of the determined content was used as an estimate of the method recovery for each allergenic ingredient on the QTM. The values resulted strictly dependent on the specific QTM and allergenic ingredient, still generally very satisfactory. Indeed, the recovery calculated on milk peptide markers resulted in 80% (m-FFV) and 87% (m-VLV), on egg markers resulted 100% (ew-ISQ) and 97% (ey-ATA), on peanut marker resulted 53% (p-TAN), on soybean marker resulted 40% (s-VLI), on hazelnut marker resulted 64% (h-ALP), and on almond 73% (a-TEE). The recoveries calculated for peanut and soybean markers resulted quite low; however, further investigation will be carried out to explain such evidence.

### Compliance with current allergen reference doses

As already mentioned, the achievement of such conversion of the RUs complies with the need to correlate the method performance characteristics to a quantitative risk assessment in evaluating the impact of cross-contamination and making decisions regarding proper allergen management and labelling. In this frame, the Voluntary Incidental Trace Allergen Labelling (VITAL) program originally created by the Allergen Bureau of Australia and New Zealand provides a useful system that has been taken into consideration also by numerous countries within the European Union [2]. In particular, the VITAL program establishes reference eliciting doses (EDs) based on clinical data available for the protection of at least 95% (ED05) or 99% (ED01) of allergic people. The last version 3.0 of the VITAL program was released in October 2019 and it set the following reference dose (mg of TAFP) for action level 1: 0.2 mg for milk, egg, and peanut, 0.5 mg for soybean, and 0.1 for hazelnut and almond. Below this threshold, no precautionary labelling statement is required and 99% of the allergic population would safely consume the food. In the absence of other official and harmonized limits issued within the European Union by relevant legal bodies, some countries, such as Germany, The Netherlands, and Belgium, issued reference doses currently recommended within their national boundaries, which are highly variable across neighbor countries (see Table 5). In 2021, the Food and Agriculture Organization of the United Nations (FAO) and World Health Organization (WHO) carried out jointly an expert consultation on risk assessment of food allergens, and recently the conclusions drawn about reviewing and establishing threshold levels of priority allergens were published (see Table 5) [40]. Focusing on the six allergens here analyzed, such document significantly raised the previously recommended thresholds for egg and peanut up to 2.0 mg, for hazelnut up to 3.0 mg, and for almond up to 1 mg (the latter being a provisional value), whereas still pending is the revised threshold for milk.

**Table 5:** Summary of the food allergen reference doses (RD) [mg total allergenic food proteins] currently recommended or issued worldwide and comparison with the quantification limit assessed by this method; conversion of LOQ in absolute total allergenic food proteins [mg] was carried out considering a portion size (PS) of 25 g for chocolate bar

Allergenic ingredient	This method LOQ [mg]	VITAL 3.0	Germany^a^	The Netherlands	Belgium	CODEX FAO-WHO Expert consultation
Milk	0.002	0.2	0.1	0.016	2	2.0
Egg	0.02^**b**^	0.2	0.03	0.0043	2	2.0
Peanut	0.004	0.2	0.2	0.015	2	2.0
Soybean	0.03	0.5	1	0.078	5	nd
Hazelnut	0.005	0.1	0.1	0.011	3	3.0
Almond	0.004	0.1	nd	nd	1	1.0^c^

^a^Based on VITAL 2.0 action levels

^b^The conversion for ew-ISQ was carried out considering the theoretical value of ovalbumin relative percentage in egg white (54%) because threshold doses refer to egg white proteins only

^c^Provisional value

For a direct comparison with these action levels, the detection limits achieved by the method under validation were converted in absolute amount, considering a portion size of 25 g reasonable for chocolate bar (see Table 5). Noteworthy, the obtained sensitivity performance is well above all the issued/recommended limits except for the Dutch legislation, which appears to be unrealistically strict compared to the other official documents.

## Conclusions

In this investigation, the in-house validation of an MS-based quantitative method for six allergens determination in chocolate matrix has been accomplished. Several performance characteristics have been described according to official guidelines, and the results were very promising in terms of selectivity and sensitivity. The quantitative information was retrieved by means of matrix-matched calibration curves and synthetic peptides as external standards, with proper conversion factors experimentally determined by discovery experiments on the six allergenic ingredients. To the best of our knowledge, this investigation represents the first attempt to provide a consistent approach for conversion factors calculation of six main allergenic ingredients. Noteworthy, the method validation has been carried out on well-characterized chocolate samples produced in a food pilot plant to mimic real samples and incurred at defined concentration levels close to the main threshold doses relevant from the clinical point of view. An in-depth discussion about the main sources of uncertainty of the analytical measurement has been provided. The sensitivity achieved was always in compliance with the various threshold doses issued or recommended worldwide, but interestingly it resulted slightly different depending on the specific allergenic ingredient. This evidence highlights one of the pitfalls of multiallergen methods; indeed, while MS can detect multiple peptides, the exhaustive extraction of proteins, with very different properties, remains very challenging. In perspective, reference method procedures are likely to be protein-dependant and different methods may be required for different proteins.

### Supplementary Information

Below is the link to the electronic supplementary material.

Supplementary file1 (DOCX 4168 KB)

## Data Availability

All data have been included in the manuscript and in the online resource.

## References

[1] Monaci L, De Angelis E, Montemurro N, Pilolli R (2018). Comprehensive overview and recent advances in proteomics MS based methods for food allergens analysis. Trends in Analytical Chem.

[2] Allergen Bureau, Summary of the 2019 VITAL Scientific Expert Panel Recommendations. 2019. https://vital.allergenbureau.net/wp-content/uploads/2021/03/VSEP-2019-Summary-Recommendations_FINAL_Sept2019.pdf. Accessed 13 Apr 2023.

[3] Martinez-Esteso MJ, O’Connor G, Nørgaard J, Breidbach A, Brohée M, Cubero-Leon E, Nitride C, Robouch P, Emons H (2020). A reference method for determining the total allergenic protein content in a processed food: the case of milk in cookies as proof of concept. Anal Bioanal Chem.

[4] Monaci L, De Angelis E, Guagnano R, Ganci AP, Garaguso I, Fiocchi A, Pilolli R (2020). Validation of a MS based proteomics method for milk and egg quantification in cookies at the lowest VITAL levels: an alternative to the use of precautionary labeling. Foods.

[5] Nelis JLD, Broadbent JA, Bose U, Anderson A, Colgrave ML (2022). Targeted proteomics for rapid and robust peanut allergen quantification. Food Chem..

[6] Parker CH, Khuda SE, Pereira M, Ross MM, Fu TJ, Fan X, Wu Y, Williams KM, DeVries J, Pulvermacher B, Bedford B, Zhang X, Jackson LS (2015). Multi-allergen quantitation and the impact of thermal treatment in industry-processed baked goods by ELISA and liquid chromatography-tandem mass spectrometry. J Agric Food Chem.

[7] Boo CC, Parker CH, Jackson LS (2018). A Targeted LC-MS/MS method for the simultaneous detection and quantitation of egg, milk, and peanut allergens in sugar cookies. J AOAC Int.

[8] Sayers RL, Gethings LA, Lee V, Balasundaram A, Johnson PE, Marsh JA, Wallace A, Brown H, Rogers A, Langridge JI, Mills ENC (2018). Microfluidic separation coupled to mass spectrometry for quantification of peanut allergens in a complex food matrix. J Proteome Res.

[9] Ramachandran B, Yang CT, Downs ML (2020). Parallel reaction monitoring mass spectrometry method for detection of both casein and whey milk allergens from a baked food matrix. J Proteome Res.

[10] Monaci L, Pilolli R, De Angelis E, Godula M, Visconti A (2014). Multi-allergen detection in food by micro high-performance liquid chromatography coupled to a dual cell linear ion trap mass spectrometry. J Chromatogr A.

[11] Torii A, Seki Y, Arimoto C, Hojo N, Iijima K, Nakamura K, Ito R, Yamakawa H, Akiyama H (2023). Development of a simple and reliable LC-MS/MS method to simultaneously detect walnut and almond as specified in food allergen labelling regulations in processed foods. Curr Res Food Sci..

[12] Pilolli R, Chaudhari R, Palmisano F, Monaci L (2017). Development of a mass spectrometry immunoassay for unambiguous detection of egg allergen traces in wines. Anal Bioanal Chem.

[13] Gavage M, Van Vlierberghe K, Dieu M, Renard P, Arnould T, De Loose M, Gevaert K, Gillard N, Van Poucke C (2022). Development and validation of a quantitative method for multiple allergen detection in food using concatemer-based isotope dilution mass spectrometry. J AOAC Int.

[14] Luparelli A, Losito I, De Angelis E, Pilolli R, Monaci L (2023). Multi-target detection of nuts and peanuts as hidden allergens in bakery products through bottom-up proteomics and high-resolution mass spectrometry. Foods.

[15] Yao K, Yang Y, Liu J, Zhang J, Shao B, Zhang Y (2021). Labeled peptide-free UHPLC-MS/MS method used for simultaneous determination of shrimp and soybean in sauce products. J Agric Food Chem.

[16] Pilolli R, De Angelis E, Monaci L (2017). Streamlining the analytical workflow for multiplex MS/MS allergen detection in processed foods. Food Chem.

[17] Pilolli R, De Angelis E, Monaci L (2018). In house validation of a high resolution mass spectrometry Orbitrap-based method for multiple allergen detection in a processed model food. Anal Bioanal Chem.

[18] Mills ENC, Adel-Patient K, Bernard H, De Loose M, Gillard N, Huet AC, Larré C, Nitride C, Pilolli R, Van Poucke C, Monaci L (2019). Detection and quantification of allergens in foods and minimum eliciting doses in food-allergic individuals (ThRAll). J AOAC Int.

[19] Worm M, Moneret-Vautrin A, Scherer K, Lang R, Fernandez-Rivas M, Cardona V, Kowalski ML, Jutel M, Poziomkowska-Gesicka I, Papadopoulos NG, Beyer K, Mustakov T, Christoff G, Bilò MB, Muraro A, Hourihane JO, Grabenhenrich LB (2014). First European data from the network of severe allergic reactions (NORA). Allergy.

[20] Turner PJ, Gowland MH, Sharma V, Ierodiakonou D, Harper N, Garcez T, Pumphrey R, Boyle RJ (2015). Increase in anaphylaxis-related hospitalizations but no increase in fatalities: an analysis of United Kingdom national anaphylaxis data, 1992–2012. J Allergy Clin Immunol.

[21] Bucchini L, Guzzon A, Poms R, Senyuva H (2016). Analysis and critical comparison of food allergen recalls from the European Union, USA, Canada, Hong Kong, Australia and New Zealand. Food Addit Contam Part A Chem Anal Control Expo Risk Assess.

[22] Huet AC, Paulus M, Henrottin J, Brossard C, Tranquet O, Bernard H, Pilolli R, Nitride C, Larré C, Adel-Patient K, Monaci L, Mills ENC, De Loose M, Gillard N, Van Poucke C (2022). Development of incurred chocolate bars and broth powder with six fully characterised food allergens as test materials for food allergen analysis. Anal Bioanal Chem.

[23] Shefcheck KJ, Callahan JH, Musser SM (2006). Confirmation of peanut protein using peptide markers in dark chocolate using liquid chromatography−tandem mass spectrometry (LC-MS/MS). J Agric Food Chem.

[24] Korte R, Brockmeyer J (2016). MRM3 -based LC-MS multi-method for the detection and quantification of nut allergens. Anal Bioanal Chem.

[25] New LS, Schreiber A, Stahl-Zeng J, Liu H-F (2018). Simultaneous analysis of multiple allergens in food products by LC-MS/MS. J AOAC Int.

[26] Planque M, Arnould T, Dieu M, Delahaut P, Renard P, Gillard N (2016). Advances in ultra high performance liquid chromatography coupled to tandem mass spectrometry for sensitive detection of several food allergens in complex and processed foodstuffs. J Chromatogr A.

[27] Gu S, Chen N, Zhou Y, Zhao C, Zhan L, Qu L, Cao C, Han L, Deng X, Ding T, Song C, Ding Y (2018). A rapid solid-phase extraction combined with liquid chromatography-tandem mass spectrometry for simultaneous screening of multiple allergens in chocolates. Food Control.

[28] Korte R, Oberleitner D, Brockmeyer J (2019). Determination of food allergens by LC-MS: impacts of sample preparation, food matrix, and thermal processing on peptide detectability and quantification. J Proteomics.

[29] New LS, Stahl-Zeng J, Schreiber A, Cafazzo M, Liu A, Brunelle S, Liu H-F (2020). Detection and quantitation of selected food allergens by liquid chromatography with tandem mass spectrometry: first action 2017.17. J AOAC Int..

[30] Henrottin J, Piolli R, Huet AC, Van Poucke C, Nitride C, De Loose M, Tranquet O, Larré C, Adel-Patient K, Bernard H, Mills ENC, Gillard N, Monaci L (2023). Optimization of a sample preparation workflow based on UHPLC-MS/MS method for multi-allergen detection in chocolate: an outcome of the ThRAll project. Food Control.

[31] Magnusson B, Örnemark U. (Eds.) Eurachem guide: the fitness for purpose of analytical methods – a laboratory guide to method validation and related topics, 2nd ed. 2014. ISBN 978–91–87461–59–0. Available from www.eurachem.org.

[32] Ellison SLR, Williams A (Eds). Eurachem/CITAC guide: quantifying uncertainty in analytical measurement, 3rd ed- 2012. ISBN 978–0–948926–30–3. Available from www.eurachem.org.

[33] Paez V, Barrett WB, Deng X, Diaz-Amigo C, Fiedler K, Fuerer C, Hostetler GL, Johnson P, Joseph G, Konings EJM, Lacorn M, Lawry J, Liu H, Marceau E, Mastovska K, Monteroso L, Pan SJ, Parker C, Phillips MM, Popping B, Radcliffe S, Rimmer CA, Roder M, Schreiber A, Sealey-Voyksner J, Shippar J, Siantar DP, Sullivan DM, Sundgaard J, Szpylka J, Turner J, Wirthwine B, Wubben JL, Yadlapalli S, Yang J, Yeung JM, Zweigenbaum J, Coates SG (2016). AOAC SMPR(®) 2016.002. J AOAC Int..

[34] European Committee for Standardization EN 17644 Foodstuffs - Detection of food allergens by liquid chromatography - mass spectrometry (LC-MS) methods - General considerations. Ref. No. EN 17644:2022. 2022. https://standards.iteh.ai/catalog/standards/cen/70bc277b-1d92-49a4-b893-c13b60eb7caf/en-17644-2022. Accessed 13 Apr 2023.

[35] Tyanova S, Temu T, Cox J (2016). The MaxQuant computational platform for mass spectrometry-based shotgun proteomics. Nat Protoc.

[36] Pilolli R, Nitride C, Gillard N, Huet AC, van Poucke C, de Loose M, Tranquet O, Larré C, Adel-Patient K, Bernard H, Mills ENC, Monaci L (2020). Critical review on proteotypic peptide marker tracing for six allergenic ingredients in incurred foods by mass spectrometry. Food Res Int..

[37] Pilolli R, Van Poucke C, De Angelis E, Nitride C, de Loose M, Gillard N, Huet AC, Tranquet O, Larré C, Adel-Patient K, Bernard H, Mills ENC, Monaci L (2021). Discovery based high resolution MS/MS analysis for selection of allergen markers in chocolate and broth powder matrices. Food Chem..

[38] MS-HOMOLOGY tool on ProteinProspector (v 6.4.5). Proteomics tools for mining sequence databases in conjunction with Mass Spectrometry experiments. https://prospector.ucsf.edu/prospector/cgi-bin/msform.cgi?form=mshomology. Accessed in Oct 2022.

[39] Andrade JM, Estévez-Pérez MG (2014). Statistical comparison of the slopes of two regression lines: A tutorial. Anal Chim Acta.

[40] Summary report of the ad hoc Joint FAO/WHO Expert Consultation on Risk Assessment of Food Allergens. Part 2: review and establish threshold levels in foods of the priority allergens. 2022 https://www.who.int/news-room/events/detail/2021/03/15/default-calendar/ad-hoc-joint-fao-who-expert-consultation-on-risk-assessment-of-food-allergens-part2-review-and-establish-threshold-levels-in-foods-of-the-priority-allergens. Accessed on 06 Apr 2023.

