# Supplementary material for: In-house validation of an LC–MS method for the multiplexed quantitative determination of total allergenic food in chocolate
Source: Anal Bioanal Chem. 2023 Aug 24;416(3):809–25. doi: 10.1007/s00216-023-04894-2 (PMC10766722; doi:10.1007/s00216-023-04894-2)
Supplement: Supplementary file 1 — Supplementary file1 (DOCX 4168 KB) [file 216_2023_4894_MOESM1_ESM.docx]

**SUPPLEMENTARY INFORMATION OF**

In-house validation of an LC-MS method for the multi-plexed quantitative determination of total allergenic food in chocolate

Rosa Pilolli ^1,^*, Antonella Lamonaca ^1,2^, Chiara Nitride ^3,4^, Elisabetta De Angelis ^1^, Christof van Poucke ^5^, Nathalie Gillard ^6^, Anne-Catherine Huet ^6^, Marc De Loose ^5^, Jean Henrottin ^6^, E.C.N. Mills ^4^ and Linda Monaci ^1^

1 Institute of Sciences of Food Production, National Research Council of Italy (ISPA-CNR), Via Giovanni Amendola 122/O, 70126 Bari, Italy.

2 University of Bari Department of Soil Plant and Food Science, Via Giovanni Amendola 165/A, 70126 Bari Italy

3 Department of Agricultural Sciences, University of Naples Federico II, Via Università 100, 80055 Portici, Italy.

4 School of Biological Sciences, Division of Infection, Immunity and Respiratory Medicine, Manchester Academic Health Science Centre, Manchester Institute of Biotechnology, University of Manchester, UK.

5 Flanders Research Institute for Agriculture, Fisheries and Food, Brusselsesteenweg 370, 9090 Melle, Belgium.

6 CER Groupe, Rue du point du Jour, 8, 6900 Marloie, Belgium.

***** Correspondence: rosa.pilolli@ispa.cnr.it; Tel.: +390805929999

**Fig. S1.** Scheme of the optimized sample preparation procedure for multiple detection of allergenic ingredients in chocolate bar samples

**Fig. S2.** Typical chromatograms acquired for blank chocolate sample spiked with isotopically labelled peptide markers.

**Fig. S3**. Graphical representation of the contribution of each source of uncertainty ($u_{ss,rel}$, $u_{MM,rel}$, $u_{CF,rel}$, $u_{RL,rel},$ $u_{PR,rel}$) to the overall standard uncertainty ($u_{x_{0}, rel}$) calculated for each incurred chocolate bar (ICB) sample analysed. For comparative purposes, all the uncertainties were plotted as relative percentages ($u_{i,rel}$).

**Table S1.** Full list of transitions acquired for marker peptides in their native and isotopically labelled forms and optimized instrumental parameters for a QSight® 220 TQ (Perkin Elmer).

| ***Allergenic Ingredient*** | ***Peptide***  ***(retention time)*** | ***Precursor (charge) /Transition (charge)*** | ***Transition*** | **Entrance voltage (EV)** | **Voltage on flat lens (CCL2)** | **Collision Energy voltage (CC)** |
| --- | --- | --- | --- | --- | --- | --- |
| *Milk Caseinate* | *mc-FFV*  *(25,19±0,19 min)* | *692,9 (+2)/920,5 (+1)* | *y8* | 32 | -156 | -27 |
|  |  | *692,9 (+2)/991,5 (+1)* | *y9* | 33 | -164 | -26 |
|  |  | *692,9 (+2)/676,4 (+1)* | *y6* | 35 | -228 | -28 |
|  |  | *692,9 (+2)/823,4 (+1)* | *y7* | 30 | -168 | -28 |
|  | *mc-FFV-L*  *(25,19±0,19 min)* | *696,9 (+2)/928,5 (+1)* | *y8* | 32 | -156 | -27 |
|  |  | *696,9 (+2)/999,5 (+1)* | *y9* | 33 | -164 | -26 |
|  |  | *696,9 (+2)/684,4 (+1)* | *y6* | 35 | -228 | -28 |
|  |  | *696,9 (+2)/831,4 (+1)* | *y7* | 30 | -168 | -28 |
|  | *mc-NAV*  *(11,31±0,11 min)* | *598,3 (+2)/911,5 (+1)* | *y8* | 22 | -180 | -24 |
|  |  | *598,3 (+2)/285,2 (+1)* | *b3* | 20 | -100 | -23 |
|  |  | *598,3 (+2)/701,4 (+1)* | *y6* | 20 | -180 | -32 |
|  |  | *598,3 (+2)/600,3 (+1)* | *y5* | 19 | -128 | -36 |
|  | *mc-NAV-L*  *(11,31±0,11 min)* | *603,3 (+2)/921,5 (+1)* | *y8* | 22 | -180 | -24 |
|  |  | *603,3 (+2)/285,2 (+1)* | *b3* | 20 | -100 | -23 |
|  |  | *603,3 (+2)/711,4 (+1)* | *y6* | 20 | -180 | -32 |
|  |  | *603,3 (+2)/610,4 (+1)* | *y5* | 19 | -128 | -36 |
| *Milk whey* | *mw-VLV*  *(13,25±0,12 min)* | *533,3 (+2)/853,4 (+1)* | *y7* | 27 | -160 | -21 |
|  |  | *533,3 (+2)/754,4 (+1)* | *y6* | 26 | -268 | -30 |
|  |  | *533,3 (+2)/641,3 (+1)* | *y5* | 30 | -156 | -17 |
|  |  | *533,3 (+2)/966,5 (+1)* | *y8* | 23 | -208 | -30 |
|  | *mw-VLV-L*  *(13,25±0,12 min)* | *537,3 (+2)/861,4 (+1)* | *y7* | 27 | -160 | -21 |
|  |  | *537,3 (+2)/762,4 (+1)* | *y6* | 26 | -268 | -30 |
|  |  | *537,3 (+2)/649,3 (+1)* | *y5* | 30 | -156 | -17 |
|  |  | *537,3 (+2)/974,5 (+1)* | *y8* | 23 | -208 | -30 |
|  | *mw-IDA*  *(2,90±0,03 min)* | *458,7 (+2)/688,4 (+1)* | *y6* | 34 | -204 | -23 |
|  |  | *458,7 (+2)/504,2 (+1)* | *y4* | 33 | -104 | -12 |
|  |  | *458,7 (+2)/617,3 (+1)* | *y5* | 34 | -320 | -30 |
|  |  | *458,7 (+2)/803,4 (+1)* | *y7* | 35 | -152 | -5 |
|  | *mw-IDA-L*  *(2,90±0,03 min)* | *462,7 (+2)/696,4 (+1)* | *y6* | 34 | -204 | -23 |
|  |  | *462,7 (+2)/512,3 (+1)* | *y4* | 33 | -104 | -12 |
|  |  | *462,7 (+2)/625,3 (+1)* | *y5* | 34 | -320 | -30 |
|  |  | *462,7 (+2)/811,4 (+1)* | *y7* | 35 | -152 | -5 |
| *Egg white* | *ew-GGL*  *(15,63±0,15 min)* | *844,4 (+2)/1331,7 (+1)* | *y12* | 37 | -220 | -30 |
|  |  | *844,4 (+2)/860,4 (+1)* | *y8* | 38 | -200 | -30 |
|  |  | *844,4 (+2)/1121,5 (+1)* | *y10* | 35 | -250 | -31 |
|  |  | *844,4 (+2)/1007,5 (+1)* | *y9* | 48 | -252 | -31 |
|  | *ew-GGL-L*  *(15,63±0,15 min)* | *849,4 (+2)/1341,7 (+1)* | *y12* | 37 | -220 | -30 |
|  |  | *849,4 (+2)/870,4 (+1)* | *y8* | 38 | -200 | -30 |
|  |  | *849,4 (+2)/1131,5 (+1)* | *y10* | 35 | -250 | -31 |
|  |  | *849,4 (+2)/1017,5 (+1)* | *y9* | 48 | -252 | -31 |
|  | *ew-ISQ*  *(4,4±0,2 min)* | *592 (+3)/859,4 (+1)* | *y8* | 30 | -148 | -29 |
|  |  | *592 (+3)/788,4 (+1)* | *y7* | 40 | -200 | -25 |
|  |  | *592 (+3)/778,4 (+1)* | *b8* | 37 | -185 | -25 |
|  |  | *592 (+3)/659,3 (+1)* | *y6* | 41 | -200 | -26 |
|  | *ew-ISQ-L*  *(4,4±0,2 min)* | *595,3 (+3)/869,4 (+1)* | *y8* | 30 | -148 | -29 |
|  |  | *595,3 (+3)/798,4 (+1)* | *y7* | 40 | -200 | -25 |
|  |  | *595,3 (+3)/778,4 (+1)* | *b8* | 37 | -185 | -25 |
|  |  | *595,3 (+3)/669,4 (+1)* | *y6* | 41 | -200 | -26 |
|  | *ew-ISQ*  *(4,4±0,2 min)* | *887,5 (+2)/996,5 (+1)* | *y9* | 79 | -312 | -52 |
|  |  | *887,5 (+2)/1067,5 (+1)* | *y10* | 50 | -348 | -47 |
|  |  | *887,5 (+2)/1138,6 (+1)* | *y11* | 77 | -252 | -49 |
|  | *ew-ISQ-L*  *(4,4±0,2 min)* | *892,5 (+2)/1006,5 (+1)* | *y9* | 79 | -312 | -52 |
|  |  | *892,5 (+2)/1077,5 (+1)* | *y10* | 50 | -348 | -47 |
|  |  | *892,5 (+2)/1148,6 (+1)* | *y11* | 77 | -252 | -49 |
| *Egg yolk* | *ey-ATA*  *(18,37±0,16 min)* | *637,3 (+2)/931,5 (+1)* | *y7* | 35 | -160 | -26 |
|  |  | *637,3 (+2)/731,4 (+1)* | *y5* | 35 | -200 | -37 |
|  |  | *637,3 (+2)/844,5 (+1)* | *y6* | 27 | -272 | -26 |
|  |  | *637,3 (+2)/785,4 (+1)* | *b8* | 35 | -156 | -16 |
|  | *ey-ATA-L*  *(18,37±0,16 min)* | *642,4 (+2)/941,5 (+1)* | *y7* | 35 | -160 | -26 |
|  |  | *642,4 (+2)/741,4 (+1)* | *y5* | 35 | -200 | -37 |
|  |  | *642,4 (+2)/854,5 (+1)* | *y6* | 27 | -272 | -26 |
|  |  | *642,4 (+2)/785,4 (+1)* | *b8* | 35 | -156 | -16 |
|  | *ey-NIG*  *(9,07±0,10 min)* | *479,8 (+2)/731,4 (+1)* | *y7* | 23 | -170 | -14 |
|  |  | *479,8 (+2)/545,3 (+1)* | *y5* | 20 | -140 | -31 |
|  |  | *479,8 (+2)/674,4 (+1)* | *y6* | 23 | -124 | -15 |
|  |  | *479,8 (+2)/844,5 (+1)* | *y8* | 22 | -144 | -24 |
|  | *ey-NIG-L*  *(9,07±0,10 min)* | *483,8 (+2)/739,4 (+1)* | *y7* | 23 | -170 | -14 |
|  |  | *483,8 (+2)/553,3 (+1)* | *y5* | 20 | -140 | -31 |
|  |  | *483,8 (+2)/682,4 (+1)* | *y6* | 23 | -124 | -15 |
|  |  | *483,8 (+2)/852,5 (+1)* | *y8* | 22 | -144 | -24 |
| *Peanut* | *p-SPD*  *(10,64±0,11 min)* | *695,4 (+2)/700,4 (+1)* | *y7* | 43 | -200 | -34 |
|  |  | *695,4 (+2)/814,4 (+1)* | *y8* | 33 | -212 | -28 |
|  |  | *695,4 (+2)/977,5 (+1)* | *y9* | 30 | -200 | -25 |
|  |  | *695,4 (+2)/1090,6 (+1)* | *y10* | 30 | -210 | -26 |
|  | *p-SPD-L*  *(10,64±0,11 min)* | *699,4 (+2)/708,4 (+1)* | *y7* | 43 | -200 | -34 |
|  |  | *699,4 (+2)/822,5 (+1)* | *y8* | 33 | -212 | -28 |
|  |  | *699,4 (+2)/985,5 (+1)* | *y9* | 30 | -200 | -25 |
|  |  | *699,4 (+2)/1098,6 (+1)* | *y10* | 30 | -210 | -26 |
|  | *p-TAN*  *(20,62±0,17 min)* | *628,4 (+2)/741,5 (+1)* | *y6* | 35 | -188 | -31 |
|  |  | *628,4 (+2)/854,6 (+1)* | *y7* | 35 | -176 | -29 |
|  |  | *628,4 (+2)/1083,7 (+1)* | *y9* | 30 | -188 | -24 |
|  |  | *628,4 (+2)/969,6 (+1)* | *y8* | 35 | -116 | -32 |
|  | *p-TAN-L*  *(20,62±0,17 min)* | *633,4 (+2)/751,5 (+1)* | *y6* | 35 | -188 | -31 |
|  |  | *633,4 (+2)/864,6 (+1)* | *y7* | 35 | -176 | -29 |
|  |  | *633,4 (+2)/1093,7 (+1)* | *y9* | 30 | -188 | -24 |
|  |  | *633,4 (+2)/979,6 (+1)* | *y8* | 35 | -116 | -32 |
| *Soybean* | *s-VFD*  *(8,96±0,10 min)* | *575,3 (+2)/788,4 (+1)* | *y7* | 30 | -120 | -28 |
|  |  | *575,3 (+2)/731,4 (+1)* | *y6* | 24 | -112 | -26 |
|  |  | *575,3 (+2)/602,3 (+1)* | *y5* | 30 | -108 | -27 |
|  |  | *575,3 (+2)/903,4 (+1)* | *y8* | 30 | -120 | -27 |
|  | *s-VFD-L*  *(8,96±0,10 min)* | *580,3 (+2)/798,4 (+1)* | *y7* | 30 | -120 | -28 |
|  |  | *580,3 (+2)/741,4 (+1)* | *y6* | 24 | -112 | -26 |
|  |  | *580,3 (+2)/612,3 (+1)* | *y5* | 30 | -108 | -27 |
|  |  | *580,3 (+2)/913,4 (+1)* | *y8* | 30 | -120 | -27 |
|  | *s-VLI*  *(19,67±0,17 min)* | *713,4 (+2)/1001,6 (+1)* | *y9* | 30 | -180 | -23 |
|  |  | *713,4 (+2)/425,3 (+1)* | *b4* | 30 | -150 | -23 |
|  |  | *713,4 (+2)/776,4 (+1)* | *y7* | 25 | -210 | -44 |
|  |  | *713,4 (+2)/904,5 (+1)* | *y8* | 30 | -230 | -27 |
|  | *s-VLI-L*  *(19,67±0,17 min)* | *718,4 (+2)/1011,6 (+1)* | *y9* | 30 | -180 | -23 |
|  |  | *718,4 (+2)/425,3 (+1)* | *b4* | 30 | -150 | -23 |
|  |  | *718,4 (+2)/786,4 (+1)* | *y7* | 25 | -210 | -44 |
|  |  | *718,4 (+2)/914,5 (+1)* | *y8* | 30 | -230 | -27 |
| *Almond* | *a-TEE*  *(16,80±0,15 min)* | *718,4 (+2)/891,5 (+1)* | *y8* | 31 | -184 | -27 |
|  |  | *718,4 (+2)/744,4 (+1)* | *y7* | 34 | -184 | -31 |
|  |  | *718,4 (+2)/962,5 (+1)* | *y9* | 28 | -160 | -27 |
|  |  | *718,4 (+2)/805,4 (+1)* | *b7* | 30 | -172 | -27 |
|  | *a-TEE-L*  *(16,80±0,15 min)* | *723,4 (+2)/901,5 (+1)* | *y8* | 31 | -184 | -27 |
|  |  | *723,4 (+2)/754,4 (+1)* | *y7* | 34 | -184 | -31 |
|  |  | *723,4 (+2)/972,6 (+1)* | *y9* | 28 | -160 | -27 |
|  |  | *723,4 (+2)/805,4 (+1)* | *b7* | 30 | -172 | -27 |
|  | *a-TEE*  *(16,80±0,15 min)* | *479,2 (+2)/631,4 (+1)* | *y6* | 22 | -120 | -22 |
|  |  | *479,2 (+3)/692,3 (+1)* | *b6* | 19 | -115 | -20 |
|  |  | *479,2 (+3)/517,3 (+1)* | *y5* | 18 | -116 | -23 |
|  |  | *479,2 (+3)/545,2 (+1)* | *b5* | 18 | -110 | -19 |
|  | *a-TEE-L*  *(16,80±0,15 min)* | *482,6 (+3)/641,4 (+1)* | *y6* | 22 | -120 | -22 |
|  |  | *482,6 (+3)/692,3 (+1)* | *b6* | 19 | -115 | -20 |
|  |  | *482,6 (+3)/527,3 (+1)* | *y5* | 18 | -116 | -23 |
|  |  | *482,6 (+3)/545,2 (+1)* | *b5* | 18 | -110 | -19 |
|  | *a-ADIF*  *(10,04±0,09 min)* | *403,2 (+2)/506,3 (+1)* | *y4* | 20 | -90 | -15 |
|  |  | *403,2 (+2)/619,4 (+1)* | *y5* | 20 | -90 | -15 |
|  |  | *403,2 (+2)/734,4 (+1)* | *y6* | 20 | -92 | -18 |
|  |  | *403,2 (+2)/447,2 (+1)* | *b4* | 19 | -82 | -12 |
|  | *a-ADIF-L*  *(10,04±0,09 min)* | *408,2 (+2)/516,3 (+1)* | *y4* | 20 | -90 | -15 |
|  |  | *408,2 (+2)/629,4 (+1)* | *y5* | 20 | -90 | -15 |
|  |  | *408,2 (+2)/744,4 (+1)* | *y6* | 20 | -92 | -18 |
|  |  | *408,2 (+2)/447,2 (+1)* | *b4* | 19 | -82 | -12 |
| *Hazelnut* | *h-ADIY*  *(7,61±0,10 min)* | *576,3 (+2)/852,4 (+1)* | *y7* | 20 | -150 | -24 |
|  |  | *576,3 (+2)/689,4 (+1)* | *y6* | 20 | -148 | -31 |
|  |  | *576,3 (+2)/588,3 (+1)* | *y5* | 20 | -150 | -29 |
|  |  | *576,3 (+2)/693,3 (+1)* | *b6* | 18 | -108 | -18 |
|  | *h-ADIY-L*  *(7,61±0,10 min)* | *581,3 (+2)/862,4 (+1)* | *y7* | 20 | -150 | -24 |
|  |  | *581,3 (+2)/699,4 (+1)* | *y6* | 20 | -148 | -31 |
|  |  | *581,3 (+2)/598,3 (+1)* | *y5* | 20 | -150 | -29 |
|  |  | *581,3 (+2)/693,3 (+1)* | *b6* | 18 | -108 | -18 |
|  | *h-ALP*  *(22,03±0,18 min)* | *815,4 (+2)/906,5 (+1)* | *y8* | 30 | -216 | -31 |
|  |  | *815,4 (+2)/835,4 (+1)* | *y7* | 30 | -192 | -30 |
|  |  | *815,4 (+2)/1019,6 (+1)* | *y9* | 30 | -200 | -31 |
|  |  | *815,4 (+2)/1445,7 (+1)* | *y13* | 30 | -244 | -31 |
|  | *h-ALP-L*  *(22,03±0,18 min)* | *820,4 (+2)/916,5 (+1)* | *y8* | 30 | -216 | -31 |
|  |  | *820,4 (+2)/845,5 (+1)* | *y7* | 30 | -192 | -30 |
|  |  | *820,4 (+2)/1029,6 (+1)* | *y9* | 30 | -200 | -31 |
|  |  | *820,4 (+2)/1455,7 (+1)* | *y13* | 30 | -244 | -31 |

**Table S2.** Calculation of detection and quantification limits (LOD and LOQ) by calibration approach in the low concentration range of the matrix matched calibration curves (regression model y=bx+a): alternative approaches to estimate the standard deviation (all limits are reported in fmol/µl).

| **ALLERGENIC INGREDIENT (QUANTITATIVE MARKER)** | **Low concentration range**  **[fmol/µl]** | **Standard Deviation of Regression residuals** | | **Standard Deviation of the lowest detected point (LDP)** | |
| --- | --- | --- | --- | --- | --- |
|  |  | **LOD =** $\boldsymbol{3*}\frac{\boldsymbol{SD}_{\frac{\boldsymbol{y}}{\boldsymbol{x}}}}{\boldsymbol{b}}$ | **LOQ =** $\boldsymbol{10}\boldsymbol{*}\frac{\boldsymbol{SD}_{\frac{\boldsymbol{y}}{\boldsymbol{x}}}}{\boldsymbol{b}}$ | **LOD =** $\boldsymbol{3*}\frac{\boldsymbol{SD}_{\boldsymbol{LDP}}}{\boldsymbol{b}}$ | **LOQ =** $\boldsymbol{10}\boldsymbol{*}\frac{\boldsymbol{SD}_{\boldsymbol{LDP}}}{\boldsymbol{b}}$ |
| MILK-CASEINATE (mc-FFV) | 0,5 – 5 | 0,4 | 1,2 | 0,05 | 0,17 |
| MILK-WHEY (mw-VLV) | 0,5 - 5 | 0,4 | 1,4 | 0,06 | 0,2 |
| EGG-WHITE (ew-ISQ) | 1 - 25 | 2 | 8 | 0,6 | 1,9 |
| EGG-YOLK (ey-ATA) | 0,5 - 5 | 0,7 | 2 | 0,2 | 0,7 |
| PEANUT (p-TAN) | 0,5 - 5 | 0,3 | 1,1 | 0,19 | 0,6 |
| SOYBEAN (s-VLI) | 0,5 - 5 | 0,6 | 2 | 0,18 | 0,6 |
| HAZELNUT (h-ALP) | 0,5 - 5 | 0,7 | 2 | 0,3 | 1,1 |
| ALMOND (a-TEE) | 0,5 - 5 | 0,5 | 1,7 | 0,08 | 0,4 |

**Table S3.** Protein molar masses and conversion factors calculated according to the experimental mass spectrometry based approach (discovery experiments on the allergenic ingredients)

| **Allergenic Ingredient** | **Allergen Code/Protein** | **Peptide marker** | **Detected accessions coding for the marker** | **Protein Molar Mass (g/mol)** | | | **Conversion Factors (CF)** | | |
| --- | --- | --- | --- | --- | --- | --- | --- | --- | --- |
|  |  |  |  | **Centered Value (cMM)** | **uncertainty,** $u_{MM}$**, k=1** | **Relative** $u_{MM. rel}$ **(%)** | **Centered Value (cCF)** | **uncertainty,**  $u_{CF}$**, k=1** | **Relative** $u_{CF, rel}$ **(%)** |
| **Milk** | Bos d 9 - α-s1-Casein | mc-FFV | P02662/αS1-casein | 20802 | 1255 | 6 % | 0,374 | 0,009 | 5% |
|  | Bos d 10 - α-s2-Casein | mc-NAV | P02663/αS2-casein | 23836 | 296 | 1,2 % | 0,098 | 0,003 | 6% |
|  | Bos d 5 - β-Lactoglobulin | mw-IDA,  mw-VLV | P02754/β-lactoglobulin | 18272 | 104 | 0,6 % | 0,193 | 0,003 | 3% |
| **Egg** | Gal d 2 - Ovalbumin | ew-GGL,  ew-ISQ | P01012/Ovalbumin | 42750 | - | - | 0,468 | 0,003 | 1,2% |
|  | Gal d 6 - Ygp42 | ey-ATA | P87498/Vitellogenin-1  A0A1D5NUW2/Phosvitin | 209146 | 28 | 0,013 % | 0,0413 | 0,0004 | 2% |
|  | - | ey-NIG | P02845/Vitellogenin-2  F1NFL6/Phosvitin | 203440 | 170 | 0,08% | 0,1056 | 0,0012 | 2% |
| **Peanut** | Ara h 3 Cupin (Legumin-Type, 11S Globulin, Glucinin) | p-SPD | A0A445CPR7/Uncharacterized protein  A0A444YLX0/Uncharacterized protein  A0A445CPR0/Uncharacterized protein  A0A444YLI/Cupin type-1 domain-containing protein (truncated) | 58096 | 319 | 0,6 | 0,695 | 0,006 | 2% |
|  |  | p-TAN | A0A445CPR7/Uncharacterized protein  A0A445CPR0/Uncharacterized protein | 58296 | 119 | 0,2% | 0,550 | 0,003 | 1.2% |
| **Soybean** | Gly m 6 - Glycinin (Legumin, 11S Globulin) | s-VFD,  s-VLI | P04776 Glycinin G1 | 53624 | - | - | 0,108 | 0,005 | 8% |
| **Hazelnut** | Cor a 9 - 11S Seed Storage Globulin (Legumin-Like) | h-ADI,  h-ALP | A0A0A0P7E3/Cor a 9  Q8W1C2 11S globulin like protein | 56645 | 145 | 0,3% | 0,799 | 0,005 | 1,3% |
| **Almond** | Pru du 6 - Amandin, 11S Globulin Legumin-Like Protein | a-ADI,  a-TEE | A0A5E4FFS0 Legumin  A0A5E4FK23 Legumin | 60928 | 53 | 0,09% | 0,765 | 0,004 | 1,0% |

**Table S4**. Theoretical calculation of egg proteins conversion factors (CF) and relevant uncertainties (u, k=1). Data about protein composition of eggs, both as whole, white and yolk, have been collected and elaborated from references 1-16 to obtain the theoretical relative abundance of main egg proteins in total egg protein.

| **Feature** | **Min** | **Max** | **Center** | **Uncertainty (**$u_{i, rel}$, **k=1)** | $u_{i, rel}$ **(%)** |
| --- | --- | --- | --- | --- | --- |
| Whole egg protein content (%w/w) | 11,6 | 13,4 | 12,5 | 0,5 | 4% |
| Egg white protein content (%w/w) | 9,5 | 12,3 | 10,9 | 0,8 | 7% |
| Egg yolk protein content (%w/w) | 15 | 17,3 | 16,2 | 0,7 | 4% |
| Egg shell protein content (%w/w) | 3,3 | 6,4 | 4,9 | 0,9 | 18% |
|  | | | | | |
| % w/w egg white over whole egg^a^ | 62,765 | 71,587 | 67 | 3 | 4% |
| % w/w egg yolk over whole egg^a^ | 28,413 | 37,235 | 33 | 3 | 8% |
| % egg white proteins over total egg proteins^a^ | 48,069 | 67,384 | 58 | 6 | 10% |
| % egg yolk proteins over 100g total egg proteins^a^ | 32,616 | 51,931 | 42 | 6 | 13% |
|  | | | | | |
| % Ovalbumin in egg white protein | 54 | x | x | x | x |
| % Ovotransferrin in egg white protein | 12 | x | x | x | x |
| % Ovomucoid in egg white protein | 11 | x | x | x | x |
| % Ovomucin in egg white protein | 4 | x | x | x | x |
| % Lysozyme in egg white protein | 4 | x | x | x | x |
|  | | | | | |
| % High Density Lipoprotein, HDL (Lipovitellins) in egg yolk protein | 35 | 40 | 37,5 | 1,4 | 4% |
| % Globular proteins (Livetins) in egg yolk protein | 8 | 38 | 23 | 9 | 39% |
| % Phosphoprotein (Phosvitin) in egg yolk protein | 8 | 13 | 10,7 | 1,6 | 15% |
| % Low Density Lipoprotein, LDL (Lipovitellenin) in egg yolk protein | 17 | 49 | 32,8 | 9,1 | 28% |
|  | | | | | |
| *Ovalbumin CF in egg protein* | *0,25957* | *0,36388* | *0,31* | *0,03* | *10%* |
| *Ovotransferrin CF in egg protein* | *0,05768* | *0,08086* | *0,069* | *0,007* | *10%* |
| *Ovomucoid CF in egg protein* | *0,05288* | *0,07412* | *0,063* | *0,006* | *10%* |
| *Ovomucin CF in egg protein* | *0,01682* | *0,02358* | *0,020* | *0,002* | *10%* |
| *Lysozyme CF in egg protein* | *0,01682* | *0,02358* | *0,020* | *0,002* | *10%* |
| *HDL* (Lipovitellins) *CF in egg protein* | *0,11415* | *0,20772* | *0,16* | *0,03* | *17%* |
| *Globular proteins* (Livetins) *CF in egg protein* | *0,02446* | *0,24927* | *0,14* | *0,07* | *47%* |
| *Phosphoprotein* (Phosvitin) *CF in in egg protein* | *0,02609* | *0,06959* | *0,048* | *0,013* | *26%* |
| *LDL* (Lipovitellenin) *CF in egg protein* | *0,05545* | *0,25187* | *0,154* | *0,057* | *37%* |

*^a^ (scaled over edible portion - without shell)*

**Table S5.** Summary of the quantification results with relevant combined and expanded (k=2) uncertainty obtained with the prototype reference method on incurred chocolate samples at four concentration levels. All the determined concentrations were reported as µg of total allergenic food protein per g of chocolate (µg _TAFP_/g _food_). The conversion of the reporting units from the peptide level (fmol/µL) to µg _TAFP_/g _food_ has been calculated according to the factors reported in Table S3.

| **ICB sample** | **MILK CASEINATE** | | | | **MILK WHEY** | | | |
| --- | --- | --- | --- | --- | --- | --- | --- | --- |
|  | **mc-FFV (QTM^a^)** | | **mc-NAV (QLM^b^)** | | **mw-VLV (QTM)** | | **mw-IDA (QLM)** | |
|  | **QTT^c^** | **QLT^d^** | **QTT** | **QLT** | **QTT** | **QLT** | **QTT** | **QLT** |
| ICB 2 | 3,0 ± 1,0 | >LOQ | >LOD | >LOD | n.d. | n.d. | n.d. | n.d. |
| ICB 4 | 6 ± 2 | >LOQ | >LOQ | >LOQ | n.d. | n.d. | n.d. | n.d. |
| ICB 10 | 14 ± 5 | >LOQ | >LOQ | >LOQ | n.d. | n.d. | n.d. | n.d. |
| ICB 40 | 45 ± 17 | >LOQ | >LOQ | >LOQ | 0,9 ± 0,5 | >LOQ | >LOQ | >LOD |
| **ICB sample** | **EGG WHITE** | | | | **EGG YOLK** | | | |
|  | **ew-ISQ (QTM)** | | **ew-GGL (QLM)** | | **ey-ATA (QTM)** | | **ey-NIG (QLM)** | |
|  | **QTT** | **QLT** | **QTT** | **QLT** | **QTT** | **QLT** | **QTT** | **QLT** |
| ICB 2 | n.d. | n.d. | n.d. | n.d. | n.d. | n.d. | n.d. | n.d. |
| ICB 4 | n.d. | n.d. | n.d. | n.d. | <LOD | n.d. | n.d. | n.d. |
| ICB 10 | 3,8 ± 1,9 | >LOD | n.d. | n.d. | 50 ± 70 | >LOQ | n.d. | n.d. |
| ICB 40 | 16 ± 6 | >LOQ | >LOQ | >LOQ | 80 ± 50 | >LOQ | >LOD | >LOD |
| **ICB sample** | **PEANUT** | | | | **SOYBEAN** | | | |
|  | **p-TAN (QTM)** | | **p-SPD (QLM)** | | **s-VLI (QTM)** | | **s-VFD (QLM)** | |
|  | **QTT** | **QLT** | **QTT** | **QLT** | **QTT** | **QLT** | **QTT** | **QLT** |
| ICB 2 | n.d. | n.d. | n.d. | n.d. | <LOD | <LOD | n.d. | n.d. |
| ICB 4 | 1,2 ± 0,7 | >LOQ | >LOD | >LOQ | >LOD | >LOD | <LOD | n.d. |
| ICB 10 | 2,0 ± 1,0 | >LOQ | >LOQ | >LOQ | 9 ± 4 | >LOQ | >LOD | >LOD |
| ICB 40 | 5 ± 2 | >LOQ | >LOQ | >LOQ | 42 ± 17 | >LOQ | >LOQ | >LOQ |
| **ICB sample** | **HAZELNUT** | | | | **ALMOND** | | | |
|  | **h-ALP (QTM)** | | **h-ADIY (QLM)** | | **a-TEE (QTM)** | | **a-ADIF (QLM)** | |
|  | **QTT** | **QLT** | **QTT** | **QLT** | **QTT** | **QLT** | **QTT** | **QLT** |
| ICB 2 | 1,1 ± 0,8 | >LOQ | <LOD | <LOD | 0,9 ± 0,5 | >LOQ | >LOQ | >LOD |
| ICB 4 | 1,8 ± 0,8 | >LOQ | >LOD | >LOD | 2,0 ± 0,8 | >LOQ | >LOQ | >LOQ |
| ICB 10 | 3,7 ± 1,5 | >LOQ | >LOQ | >LOQ | 4,8 ± 1,7 | >LOQ | >LOQ | >LOQ |
| ICB 40 | 11 ± 4 | >LOQ | >LOQ | >LOQ | 17,0 ± 6 | >LOQ | >LOQ | >LOQ |

*^a^QTM: quantitative marker*

*^b^QLM: qualitative marker*

*^c^QTT: quantitative transition*

*^d^QLT: qualitative transition*

**References**

1. Cotterill OJ, Geiger GS. Egg Product Yield Trends From Shell Eggs. Poultry Sci. 1977;56:1027-1031.
2. Sugino H, Nitoda T, Juneja LR. General chemical composition of hen eggs. In Hen Eggs, Their Basic and Applied Science; Yamamoto, T., Juneja, L. R., Hatta, H., Kim, M., Eds.; CRC Press: New York, 1997; pp 13-24.
3. Ahn DU, Kim SM, Shu H. Effect of Egg Size and Strain and Age of Hens on the Solids Content of Chicken Eggs. Poultry Sci. 1997;76:914–919.
4. Cherian G, Holsonbake TB, Goeger MP. Fatty Acid Composition and Egg Components of Specialty Eggs. Poultry Sci. 2002;81:30–33.
5. Zhang Y, Guo Y, Liu F, Luo Y. Recent development of egg protein fractions and individual proteins as encapsulant materials for delivery of bioactives. Food Chem. 2023;403:134353.
6. Egg bioscience and biotechnology / [edited by] Yoshinori Mine. 2008 ISBN 978-0-470-03998-4 (cloth), WILEY-INTERSCIENCE, A JOHN WILEY & SONS, INC., PUBLICATION
7. FoodData Central (usda.gov) Available at <https://fdc.nal.usda.gov/fdc-app.html#/?query=egg>. Accessed on April 2023.
8. Abeyrathne ED, Lee HY, Ahn DU. Egg white proteins and their potential use in food processing or as nutraceutical and pharmaceutical agents--a review. Poult Sci. 2013;92:3292-3299. doi: 10.3382/ps.2013-03391.
9. Kovacs-Nolan J, Phillips M, Mine Y. Advances in the value of eggs and egg components for human health. J Agric Food Chem. 2005;53:8421-8431. doi: 10.1021/jf050964f.
10. EFSA Panel on Dietetic Products, Nutrition and Allergies (NDA). Scientific Opinion on the evaluation of allergenic foods and food ingredients for labelling purposes. EFSA J 2014;12:3894.
11. Mine Y. Recent advances in egg protein functionality in the food system. World’s Poult. Sci. J. 2002;58:31-39. https://doi.org/10.1079/WPS20020005
12. Burley RW, Cook WH. Isolation and composition of avian egg yolk granules and their constituents [alpha]- and [beta]-lipovitellins. Canadian J Biochem Phys. 1961;39:1295–1307.
13. Anton M. Egg yolk: structures, functionalities and processes. J Sci Food Agric 2013;93:2871–2880.
14. Chang C, Lahti T, Tanaka T, Nickerson MT. Egg proteins: fractionation, bioactive peptides and allergenicity. J Sci Food Agric 2018;98:5547–5558.
15. Belitz HD, Grosch W, Schieberle P, Food Chemistry, 4th revised and extended ed., 2009 ISBN 978-3-540-69933-0, e-ISBN 978-3-540-69934-7, DOI 10.1007/978-3-540-69934-7, Publisher: Springer Berlin, Heidelberg
16. Bioactive Egg Compounds, Huopalahti R, López-Fandiño R, Anton M, Schade R (Eds.), ISBN-13: 978-3-540-37883-9 Publisher: Springer Berlin Heidelberg New York.
